# Mercury(II) Coordination Compounds Based on Pyridine‐2,5‐Dicarboxylic Acid: Synthesis, Crystal Structure, Morphological Characterization, and Biological Activities

**DOI:** 10.1155/bca/3269865

**Published:** 2026-10-29

**Authors:** Fatemeh Jamshidi, Khosro Mohammadi, Payam Hayati, Somayyeh Gharibi, Arezoo Khoradmehr, Zeinab Asen, Jan Janczak

**Affiliations:** ^1^ Department of Chemistry Faculty of Nano and Bio Sciences and Technology Persian Gulf University Bushehr 75169 Iran pgu.ac.ir; ^2^ Department of Biotechnology and Life Sciences University of Insubria Via Jean Henry Dunant, 3 21100 Varese Italy uninsubria.eu; ^3^ The Persian Gulf Marine Biotechnology Research Center The Persian Gulf Biomedical Sciences Research Institute Bushehr University of Medical Sciences Bushehr Iran bpums.ac.ir; ^4^ Shamim Beh Azma Technical Inspection Company Bushehr Iran; ^5^ Institute of Low Temperature and Structure Research Polish Academy of Sciences Okólna 2 Str. 50-422 Wroclaw Poland pan.pl

**Keywords:** anticancer, antibacterial, coordination polymer, cytotoxicity, mercury, supramolecules

## Abstract

This work reports the synthesis, spectroscopic characterization, structural investigation, and biological evaluation of two new mercury(II) coordination compounds, ([HL′][Hg(HL) (μ‐Cl)_2_])_n_ (**1**) and ([LHg]_2_(μ‐HL″)_2_(μ‐Cl)_2_Hg(H_2_O)_3_)·H_2_O (**2**), with H_2_L as 2,5‐pyridinedicarboxylic acid, L′ as 2,4‐diaminopyrimidine, and HL″ as 2,4‐diamino‐6‐hydroxypyrimidine. Single‐crystal X‐ray diffraction was employed to determine the crystal and molecular structures of the compounds, while other techniques such as FTIR, NMR, PXRD, FESEM, EDS, TGA, BET, DLS, and Zeta were applied to understand their composition and physicochemical properties. Structural analysis confirmed that compound (**1**) is a one‐dimensional coordination polymer, which has been formed through μ‐Cl bridges, while compound (**2**) consists of a supramolecular structure that is stabilized by hydrogen bonding and π–π stacking interactions. The extended crystal structures are formed through coordination and further stabilized by noncovalent interactions. Both mercury(II) coordination compounds exhibited notable in vitro antibacterial and cytotoxic activities, warranting further mechanistic and toxicological evaluation before any therapeutic extrapolation, given the well‐documented systemic toxicity of mercury.

## 1. Introduction

Coordination polymers and supramolecular assemblies have drawn significant attention because they can organize small molecular building blocks into extended functional networks. Early reviews highlighted that metal ions and organic ligands are capable of self‐assembling into diverse architectures [1]. Moreover, supramolecular chemistry enables molecules to organize through tunable noncovalent interactions, allowing the construction of functional systems [2, 3]. These approaches have opened new opportunities in catalysis, gas storage, sensing, drug delivery, and biomedical applications [4, 5].

Metal‐organic compounds are particularly attractive as their geometry and bonding environment can be precisely engineered, directly influencing both structure and function [6]. Many of these compounds interact with biological systems, often displaying antibacterial and cytotoxic activities [7]. Among various ligand families, pyridine derivatives stand out because of their versatile coordination capabilities [8]. In particular, 2,5‐pyridinedicarboxylic acid (2,5‐pydc) provides multiple coordination sites through its carboxylate groups and pyridyl nitrogen atom [9], while its anionic forms (Hpydc^−^ and pydc^2−^) can participate in proton transfer processes and hydrogen‐bonded networks [10]. This versatility makes 2,5‐pydc an attractive building block for the construction of coordination polymers and supramolecular assemblies [11].

Previous studies on coordination compounds containing pyridine‐ and pyrazine‐dicarboxylate derivatives have demonstrated that the organization of the resulting crystal structures is governed not only by primary metal–ligand coordination bonds but also by secondary noncovalent interactions. Hydrogen bonding, *π*⋯*π* interactions, and other directional intermolecular contacts can contribute substantially to crystal packing and stabilization, and their energetic contributions can be important for understanding the resulting architectures [12]. In particular, systematic studies of transition‐metal complexes containing pyridine‐2,5‐dicarboxylic acid have shown that hydrogen bonds, C–H⋯*π* and *π*⋯*π* interactions, interaction synthons, and water clusters can act cooperatively in connecting coordination units and constructing the overall supramolecular crystal lattice [13].

The structural diversity of pyridine‐2,5‐dicarboxylate systems is further illustrated by studies of pyridine‐N‐oxide‐2,5‐dicarboxylic acid derivatives, in which changes in the ligand and metal environment have been associated with different coordination modes and supramolecular organizations, including extended coordination polymer architectures [14]. Related investigations of acid–base adducts based on pyridine‐2,5‐dicarboxylic acid N‐oxide have also shown that recurrent inter‐ and intramolecular hydrogen‐bonding motifs can govern the assembly of crystalline materials [15]. Furthermore, studies of Mn(II) complexes based on N‐oxidized pyridine‐2,5‐dicarboxylic acid examined both solid‐ and solution‐state behavior, emphasizing that the structural characteristics identified crystallographically should be considered together with the behavior of the complexes under solution conditions [16].

These findings are particularly relevant when structural information is used to interpret functional properties. Variations in coordination mode, dimensionality, hydrogen‐bonding patterns, *π*⋯*π* contacts, and the presence or organization of water molecules may influence crystal packing, hydration, intermolecular cohesion, and the physicochemical behavior of coordination compounds. However, the relationship between these structural characteristics and biological activity is not necessarily direct and is strongly dependent on the specific metal–ligand system and its behavior under the relevant biological conditions.

Pioneering studies in the 1990s by MacGillivray et al. [17], followed by Yaghi et al. [18], revealed unique structures and broad applications [6]. Foundational work by Lehn [19] and Stoddart [20] provided key principles for rational supramolecular design.

Mercury(II) is particularly interesting from a coordination‐chemical perspective because of its flexible coordination behavior and ability to form structurally diverse complexes and coordination polymers with carboxylate and nitrogen‐donor ligands. Previous studies have reported Hg(II) coordination compounds containing pyridinedicarboxylic acids, demonstrating that this combination can generate distinct coordination environments and extended structural architectures [21]. Mercury(II) compounds have also been investigated for antibacterial and cytotoxic effects [7, 22–24]. Moreover, related studies on pyridine‐dicarboxylate‐based metal complexes have demonstrated that structural features arising from ligand selection and coordination environment may influence biological responses, including antiproliferative behavior [25]. Nevertheless, the pronounced intrinsic toxicity and biological reactivity of mercury require such effects to be interpreted cautiously, particularly when distinguishing antibacterial or cytotoxic activity from therapeutic potential.

Despite the considerable structural chemistry of pyridine‐2,5‐dicarboxylate coordination compounds and the established coordination versatility of Hg(II), comparatively limited attention has been directed toward Hg(II)–2,5‐pydc systems in which changes in the ancillary nitrogen‐donor ligand are examined in relation to the resulting coordination dimensionality, supramolecular organization, crystal morphology, and biological behavior. In particular, a comparative investigation in which the same primary 2,5‐pydc ligand is combined with different nitrogen‐donor components provides an opportunity to examine how ligand selection is associated with changes in structural organization and whether these differences coincide with differences in biological responses.

Accordingly, the present study was designed to investigate two Hg(II) coordination compounds constructed from the same 2,5‐pyridinedicarboxylic acid platform but containing different nitrogen‐donor organic components. The resulting compounds exhibit distinct structural organizations: Compound **(1)** forms an extended one‐dimensional coordination polymer, whereas compound **(2)** adopts a discrete trinuclear supramolecular architecture. This structural contrast provides a useful comparative framework for examining the influence of ancillary‐ligand selection on the Hg(II) coordination environment, crystal packing, dimensionality, hydrogen‐bonding and hydration patterns, and crystal morphology.

The novelty of the present work therefore does not rest solely on the determination of two previously unreported crystal structures. Rather, the study combines single‐crystal structural analysis, supramolecular‐interaction assessment, crystal morphology characterization, and biological evaluation to explore a preliminary structure–property and structure–activity relationship within a closely related pair of Hg(II)–2,5‐pydc systems. In particular, the comparison allows the effects associated with changing the ancillary nitrogen‐donor component to be considered alongside changes in coordination dimensionality and supramolecular organization. Because the present experimental design does not independently determine the relative contributions of the intact coordination compounds, free ligands, or potentially released Hg(II) species under biological assay conditions, the observed structure–activity relationships are regarded as associative rather than definitive mechanistic correlations.

The focus of this study was mercury(II), because of its unique coordination chemistry and reported biological reactivity [21–24]. Accordingly, two Hg(II) coordination compounds were designed and synthesized using 2,5‐pyridinedicarboxylic acid as the primary ligand together with different nitrogen‐containing organic components. The influence of these organic components on the coordination environment, crystal packing, supramolecular organization, and biological properties was subsequently investigated. Crystal morphology and growth were evaluated using the BFDH model in VESTA, revealing consistent structural trends. Biological evaluations further showed antibacterial and cytotoxic effects for both compounds, with differences in biological responses between the two systems. These results were considered together with their distinct crystal architectures and morphological characteristics to provide a comparative assessment of the relationship between ligand selection, structural organization, and biological behavior.

Overall, the objective of this work was to synthesize and comprehensively characterize two novel Hg(II) coordination compounds based on 2,5‐pyridinedicarboxylic acid and different nitrogen‐donor ligands, and to investigate how ligand selection is associated with their coordination structures, intermolecular interactions, crystal morphology, and biological properties. The study thereby aims to contribute to a more integrated understanding of structure–property relationships in Hg(II)–pyridinedicarboxylate coordination systems while recognizing the limitations associated with assigning the observed biological effects to a specific molecular species.

## 2. Experimental

### 2.1. Materials

2,5‐Pyridinedicarboxylic acid (H_2_L), 2,4‐diaminopyrimidine (L′), and 2,4‐diamino‐6‐hydroxypyrimidine (HL″) were purchased from Exir Chemical Company. Mercury(II) chloride and acetonitrile were obtained from Merck KGaA. The methanol was prepared from Zagros Petrochemical.

### 2.2. Synthesis of the Compounds

#### 2.2.1. Synthesis of ([HL′][Hg(HL) (μ‐Cl)_2_])_n_ (**1**) as Single Crystal

2,5‐Pyridinedicarboxylic acid (30 mg, 0.18 mmol) was dissolved in MeOH (20 mL) and added to 2,4‐diaminopyrimidine (20 mg, 0.18 mmol) dissolved in MeOH (10 mL), followed by the dropwise addition of HgCl_2_ (100 mg, 0.36 mmol) dissolved in MeOH (20 mL). The reaction mixture was refluxed for approximately 7 h at 60°C and then cooled to room temperature. Some yellow crystals were obtained by leaving the solution to evaporate at room temperature for 1 week, washing with water, and drying at room temperature.

#### 2.2.2. Synthesis of ([HL′][Hg(HL) (μ‐Cl)_2_])_n_ (**1′**) as Powder Sample of Coordination Compound (**1**)

To synthesize the powder sample of coordination compound (**1**) by the sonochemical process, a high‐density ultrasonic bath was immersed directly into the solution of a mixture of 2,5‐pyridinedicarboxylic acid (30 mg, 0.18 mmol) in MeOH (20 mL) and 2,4‐diaminopyrimidine (20 mg, 0.18 mmol) in MeOH (10 mL). At the same time, ultrasonic waves were applied to the solution containing ligands. To this solution, HgCl_2_ (100 mg, 0.36 mmol) in MeOH (20 mL) was added in a dropwise manner. The solution was irradiated by sonochemistry with a power of 60 W and a temperature of 50°C for 30 min. The precipitated powder was filtered, washed with water, and then dried at room temperature.

#### 2.2.3. Synthesis of ([LHg]_2_(μ‐HL″)_2_(μ‐Cl)_2_Hg(H_2_O)_3_)·H_2_O (**2**) as Single Crystal

The compound (**2**) was prepared by employing the hydrothermal method. A solution of 2,5‐pyridinedicarboxylic acid (167 mg, 1 mmol) in acetonitrile/distilled water (15/5 mL/mL) was added to 2,4‐diamino‐6‐hydroxypyrimidine (126 mg, 1 mmol) in distilled water solution (15 mL), followed by the addition of HgCl_2_ (270 mg, 1 mmol) in distilled water solution (20 mL). Then, the reaction mixture was transferred into a Teflon‐lined autoclave and heated at 100°C in an oven. After 60 h, the reaction was slowly cooled to room temperature under ambient conditions. After 1 week, colorless crystals suitable for X‐ray diffraction analysis were produced from the reaction solution, washed with water, and dried at room temperature.

#### 2.2.4. Synthesis of ([LHg]_2_(μ‐HL″)_2_(μ‐Cl)_2_Hg(H_2_O)_3_)·H_2_O (**2′**) as Powder Sample of Coordination Compound (**2**)

The powder sample of coordination compound (**2**) was synthesized by a sonochemical process. A high‐density ultrasonic bath was immersed directly into the solution of the mixture of 2,5‐pyridinedicarboxylic acid (167 mg, 1 mmol) in acetonitrile/distilled water (15/5 mL/mL) and 2,4‐diamino‐6‐hydroxypyrimidine (126 mg, 1 mmol) in distilled water (15 mL). Then, HgCl_2_ (270 mg, 1 mmol) in a distilled water solution (20 mL) was added dropwise to the previous solution. The solution was irradiated by sonochemistry with the power of 60 W and a temperature of 50°C for 30 min. The precipitated powders were collected, washed successively with water, and air‐dried.

### 2.3. Characterization Instruments

An ultrasonic bath (Model vCLEAN) was used for the experiments. D8 Advance X‐ray diffractometer (Bruker AXS, Germany) equipped with Cu Kα radiation was used to record the powder X‐ray diffraction (PXRD) patterns. Fourier transform infrared (FTIR) spectra for the KBr pellet were recorded using a type A One FT/IR‐4600 (Jasco, Japan) spectrometer. TGA curves were obtained from a STA 6000 thermal analyzer (PerkinElmer, USA). Nuclear magnetic resonance (NMR) spectra were taken with a 400 MHz NMR spectrometer belonging to the Avance III model. The samples were characterized by field emission scanning electron microscopy (FE‐SEM MIRA3, TESCAN, Czech Republic) after a gold coating to allow better imaging resolution. The surface area was determined via nitrogen adsorption–desorption isotherms at 77 K using a Micromeritics ASAP 2020 analyzer (Micromeritics Instrument Corporation, USA), with the Brunauer–Emmett–Teller (BET) method applied for calculation. The hydrodynamic particle size distribution and zeta potential were measured using a Nano DS analyzer (CAD Instruments, France) based on dynamic light scattering (DLS) and electrophoretic light scattering (ELS), respectively.

#### 2.3.1. X‐Ray Crystallography

Compounds (**1**) and (**2**) were characterized by single‐crystal X‐ray diffraction (SCXRD) at room temperature using clear, well‐formed crystals that provided high‐quality diffraction patterns. Data were collected on an Xcalibur four‐circle *κ* geometry diffractometer with an Atlas two‐dimensional area CCD detector, employing MoKα radiation (*λ* = 0.71073 Å) and a graphite monochromator. Unit‐cell determination and data acquisition were performed using the CrysAlisPro software (Version 1.171.42.93a, Rigaku Oxford Diffraction, 2023) and refinement of the unit‐cell parameters with the CrysAlisPro software [26].

The structures were initially solved using direct methods with SHELXT, which successfully located most nonhydrogen atomic positions. Absorption corrections were applied using spherical harmonics in the SCALE3 ABSPACK algorithm to enhance the precision of the datasets. Subsequent refinement was carried out with SHELXL2018/3, assigning anisotropic thermal displacement parameters to all nonhydrogen atoms to produce reliable and well‐refined structural models. Details of the data collection parameters, crystallographic data, and final agreement parameters are collected in Table 1. For structural analysis and visualization, molecular graphics were also produced using TOPOSPro [27] and Diamond 3.0 [28], while the external crystal morphologies were predicted with the BFDH model in VESTA [29]. Hirshfeld surfaces and fingerprint plots were generated using CrystalExplorer [30, 31], allowing detailed insight into the intermolecular interactions controlling the packing arrangements of both compounds.

**TABLE 1:** Crystal data and structure refinement parameters for (**1**) and (**2**).

Crystal data	Compound (1)	Compound (2)
Chemical formula	C_7_H_4_Cl_2_HgNO_4_·C_4_H_7_N_4_	2 (C_11_H_9_HgN_5_O_5_)·Cl_2_Hg·5 (H_2_O)
*M*_*r*_	548.74	1345.21
Crystal system, space group	Monoclinic, *P*2_1_/*c*	Monoclinic, *I*2/*a*
Temperature (K)	295	295
*a*, *b*, *c* (Å)	3.8456 (4), 30.369 (3), 12.9078 (14)	13.8189 (8), 12.8900 (6), 19.5674 (10)
β (°)	91.298 (9)	108.599 (6)
*V* (Å^3^)	1507.1 (3)	3303.4 (3)
*Z*	4	4
Radiation type	Mo *K*α	Mo *K*α
*μ* (mm^−1^)	10.6	14.16
Crystal size (mm)	0.39 × 0.05 × 0.04	0.32 × 0.09 × 0.07

*Data collection*		
Diffractometer	Xcalibur, Atlas	Xcalibur, Atlas
Absorption correction	Multi‐scan	Multi‐scan
*T*_min_, *T*_max_	0.640, 1.000	0.622, 1.000
No. of measured, independent, and observed [*I* > 2*σ* (*I*)] reflections	25691, 3840, 2417	27605, 3782, 2394
*R*_int_	0.076	0.11
(sin *θ*/*λ*)_max_ (Å^−1^)	0.691	0.65

*Refinement*		
*R* [*F* ^2^ > 2*σ* (*F* ^2^)]^a^, *wR* (*F* ^2^)^a^, *S*	0.064, 0.155, 1.00	0.051, 0.072, 1.00
No. of reflections	3840	3782
No. of parameters	209	243
Δρ_max_, Δρ_min_ (e Å^−3^)	1.83, −1.78	1.27, −0.96
CCDC No.	2353705	2353564

^a^ *R* = Σ ||*F*_*o*_|–|*F*_*c*_||/Σ*F*_*o*_, *w* *R* = {Σ [*w* ($F_{o}^{2}$–$F_{c}^{2}$)^2^]/Σ$wF_{o}^{4}$}^½^; *w* ^−1^ = *σ* ^2^ ($F_{o}^{2}$) + (*aP*)^2^ + b*P* where *P* = ($F_{o}^{2}$ + 2$F_{c}^{2}$)/3. The *a* and *b* parameters are 0.0652 and 1.512 for (**1**) and 0.0071 and 0.0 for **(2)**.

### 2.4. Biological Studies

#### 2.4.1. Antimicrobial Assays

##### 2.4.1.1. Disk Diffusion Methodology

The disk diffusion methodology, as delineated by CLSI, M100‐S22, was employed to evaluate the antibacterial efficacy of powder samples of coordination compounds (**1′**) and (**2′**) against a spectrum of seven Gram‐positive and nine Gram‐negative bacterial strains at a concentration of 5 mg/mL in a DMSO solution (refer to Table 2). This concentration (5 mg/mL) corresponds to the initial stock solution used for disk loading and does not represent the effective active concentration; the actual antibacterial potency of the compounds, expressed as MIC and MBC values in the low‐microgram‐per‐milliliter range, is presented in Table 3. Sterile 6‐mm paper disks were impregnated with 25 μL of each compound solution and placed onto the inoculated agar plates. The Gram‐positive bacterial strains were controlled with penicillin disks (10 μg), while Gram‐negative strains were regulated with ciprofloxacin disks (5 μg). Deionized water was utilized as a negative control, and the absence of antibacterial properties of the DMSO solvent on bacterial growth was also assessed. A solitary colony was incubated overnight in Brain Heart Infusion (BHI) broth (Merck, Germany), achieving turbidity conforming to the 0.5 McFarland standard to produce bacterial suspensions. For bacterial cultivation, either Merck, Germany’s Mueller–Hinton Agar, or Mueller–Hinton Agar supplemented with 5% sheep blood (specifically for *Streptococcus* spp.) was utilized. Employing sterile cotton swabs, the resultant bacterial suspensions were uniformly distributed across the agar plates. The plates were subsequently incubated for a duration of 24 h at 37°C, either in a candle jar (designated for *Streptococcus* spp.) or under standard atmospheric conditions. Each experimental trial was performed in technical duplicate (two disks per compound on the same agar plate, inoculated from a single bacterial suspension), and the diameter of the inhibition zone surrounding the well was meticulously measured using a ruler [23, 32].

**TABLE 2:** Clinical and standard bacteria lists used in this study.

Gram‐positive bacteria	Gram‐negative bacteria
*Staphylococcus aureus* ATCC 6538	*Pseudomonas aeruginosa* ATCC 15442
*Staphylococcus epidermidis* ATCC 14990	*Klebsiella pneumoniae* ATCC 13883
Methicillin‐resistant *Staphylococcus aureus* (MRSA) clinical	*Pseudomonas aeruginosa clinical* (PAO1)
*Enterococcus faecalis* ATCC 1394	*Vibrio cholerae* clinical NAG
*Streptococcus pyogenes* ATCC 19615	*Proteus mirabilis* ATCC 4371
*Streptococcus agalactiae* PTCC 1768	*Escherichia coli* ATCC 8739
*Bacillus cereus* ATCC 11778	*Shigella flexneri* ATCC 12022
	*Acinetobacter baumannii* ATCC 19606
	*Salmonella typhi* ATCC 14029 PTCC 1609

**TABLE 3:** Antimicrobial efficacy of compounds (**1′**) and (**2′**) was evaluated using the disk diffusion and broth microdilution techniques.

Bacteria	Antimicrobial assay						
	Disk diffusion method (mm^1^)			Broth microdilution method (μg/mL)			
	(**1′**)	(**2′**)	Control^2^	(**1′**)		(**2′**)	
				MIC	MBC	MIC	MBC
*Staphylococcus aureus* ATCC 6538	26.5	35.5	40	0.48	0.48	1.95	1.95
MRSA^3^ clinical	17	27	0	2.44	2.44	2.44	2.44
*Staphylococcus epidermidis* ATCC 14990	20.5	26	42	2.44	2.44	0.48	0.48
*Enterococcus faecalis* ATCC 1394	10	10.5	20	0.48	1.95	0.48	1.95
*Streptococcus pyogenes* ATCC 19615	9.5	10	25	0.12	0.12	0.12	0.24
*Streptococcus agalactiae* PTCC 1768	10	11	35	0.48	0.48	0.12	0.24
*Bacillus cereus* ATCC 11778	24.5	30	0	0.97	0.97	0.48	0.48
*Klebsiella pneumoniae* ATCC 13883	24.5	30.5	35	0.97	0.97	1.95	1.95
*Pseudomonas aeruginosa* clinical (PAO1)	23.5	25.5	30	0.97	0.97	0.48	0.48
*Pseudomonas aeruginosa* ATCC 15442	12.5	24.5	35	9.76	9.76	4.88	4.88
*Vibrio cholerae* clinical NAG	21.5	28.5	35	0.97	0.97	0.48	0.48
*Proteus mirabilis* ATCC 4371	25	30	35	0.97	0.97	0.48	0.48
*Escherichia coli* ATCC 8739	19	26.5	35	1.22	1.22	0.48	0.48
*Shigella flexneri* ATCC 12022	25.5	32.5	35	0.48	0.48	0.48	0.48
*Acinetobacter baumannii* ATCC 19606	23.5	29	40	0.97	0.97	0.48	0.48
*Salmonella typhi* ATCC 14029	23.5	29	38	0.48	0 48	0.48	0.48

^1^millimeter.

^2^Penicillin (10 μg) for Gram‐positive or ciprofloxacin (5 μg) for Gram‐negative were used as a positive control, and DMSO and DDW were used as a negative control in the disk diffusion method.

^3^Methicillin‐resistant *Staphylococcus aureus* (MRSA).

##### 2.4.1.2. Broth Microdilution Technique

The standard broth microdilution method according to CLSI M100‐S22 was employed to determine the minimum inhibitory concentration (MIC) and minimum bactericidal concentration (MBC) of compounds (**1′**) and (**2′**). Bacterial cultures were cultivated overnight at 37°C in Mueller–Hinton Broth (Merck, Germany) and adjusted to a 0.5 McFarland standard in fresh, sterile Mueller–Hinton broth to calculate the MIC. A 96‐well microtiter plate was then filled with 100 μL of Mueller–Hinton broth in each well. Serial twofold dilutions of each compound (initial concentration 5 mg/mL) were prepared in Mueller–Hinton broth before the bacterial suspension was added to each well. About 100 μL of each bacterial solution (diluted 1:150 in 0.9% saline) was introduced to each well after (**1ʹ**) particle at a concentration of 5 mg/mL was serially diluted throughout the wells. The microplates were incubated at 37°C for a period of 24 h. Each assay was performed in technical duplicate (two wells per concentration within the same microtiter plate, using a single bacterial suspension [23, 33, 34]. Table 2 shows the clinical and standard bacterial strains used in the studies, including both Gram‐positive and Gram‐negative bacteria. ATCC and PTCC designations are provided where applicable.

#### 2.4.2. Cell Culture

VERO (African green monkey kidney epithelial) and OVCAR3 (human ovarian carcinoma, female) cell lines were obtained from the Iranian Biological Resource Center and the Stem Cell Biology Research Center (Yazd, Iran) in [SEP, 2024]. Formal short tandem repeat (STR) profiling was not conducted for these cell lines in this study. The cells were sourced directly from reputable cell banks and used at low passage numbers. Routine mycoplasma testing was not performed; the absence of such testing is acknowledged as a limitation of the study. Cells were propagated in T25 flasks, utilizing a complete growth medium formulated with Dulbecco’s modified Eagle Medium (DMEM) as its base. This medium was supplemented with 10% fetal bovine serum (FBS) to provide necessary growth factors, 1% sodium bicarbonate to maintain physiological pH, and 1% penicillin–streptomycin (pen–strep) to prevent microbial contamination. To ensure consistent nutrient availability and waste removal, the culture medium was replaced every two to 3 days, thereby supporting optimal cell growth and viability.

#### 2.4.3. MTT Assay

Upon reaching approximately 80% confluence in 25‐cm^2^ flasks, cells were subcultured. The existing medium was removed, and cells were washed with 1x PBS. Detachment was achieved using 0.025% Trypsin–EDTA, with a 3‐min incubation at 37°C. Trypsin activity was then quenched by adding a complete culture medium. The cell suspension was centrifuged at 3000 rpm for 5 min, the supernatant was discarded, and the cell pellet was resuspended in fresh medium. For the experimental setup, 1 × 10^4^ cells were seeded into each well of 96‐well plates and allowed to recover for 24 h in DMEM supplemented with 10% FBS. Following recovery, the medium was replaced with fresh DMEM containing 10% FBS and varying concentrations (0.98–1000 μg/mL) of the compounds. After a 72‐h incubation, cell viability was determined using an MTT assay. Formazan crystals, formed after a 4‐h incubation with MTT, were dissolved in DMSO, and absorbance was measured at 570 nm using an ELISA reader. Absorbance values were directly proportional to the number of viable cells [23, 35].

#### 2.4.4. Statistical Analysis

Statistical analyses were conducted using IBM SPSS Statistics 22 and GraphPad Prism (Version 7.0a). To evaluate differences between control and experimental groups, a one‐way ANOVA was employed, followed by a post hoc Tukey test to identify specific pairwise differences. Data are presented as the mean ± standard deviation of the mean (SD). Statistical significance was defined as *p* < 0.05, indicating results unlikely to have occurred by chance.

## 3. Results and Discussion

### 3.1. Synthetic Perspective

The reaction between 2,5‐pyridinedicarboxylic acid and 2,4‐diaminopyrimidine compounds and HgCl_2_ produced a novel coordination compound crystal material with the formula ([HL′][Hg(HL) (μ‐Cl)_2_])_n_ (**1**). Furthermore, the combination of 2,5‐pyridinedicarboxylic acid and 2,4‐diamino‐6‐hydroxypyrimidine compounds with HgCl_2_ resulted in a new coordination compound crystal material with the formula ([LHg]_2_(μ‐HL″)_2_(μ‐Cl)_2_Hg(H_2_O)_3_)·H_2_O (**2**). Reflux treatment obtained single crystals of compound (**1**), while compound (**2**) was synthesized using hydrothermal methods (Scheme 1). In addition, compounds (**1′**) and (**2′**) were synthesized using ultrasonic irradiation techniques in a different experiment.

**Scheme 1:**
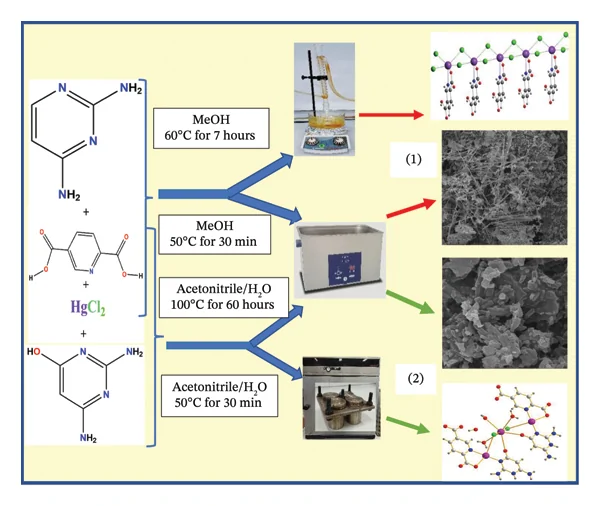
The synthetic routes of compounds (**1**) and (**2**).

### 3.2. Single‐Crystal X‐Ray Structural Analysis

#### 3.2.1. Crystal Structure and Supramolecular Features of Compound (**1**)

SCXRD analysis was used to determine the solid‐state structures of both mercury(II) coordination compounds (**1**) and (**2**). Compound (**1**) crystallizes in the monoclinic crystal system with the P2_1_/c space group, whereas compound (**2**) crystallizes in the monoclinic crystal system with the I2/a space group. Although the asymmetric unit of compound (**1**) contains one Hg(II) ion, one deprotonated 2,5‐pyridinedicarboxylate (HL^−^) ligand, one protonated 2,4‐diaminopyrimidine ([HL′]^+^), and two chloride ions, the crystal structure is extended through the interconnection of adjacent Hg(II) centers by μ‐Cl bridges, generating an infinite one‐dimensional coordination polymer. The Hg(II) center exhibits a six‐coordinate environment composed of four μ‐Cl bridging chloride donors with different Hg–Cl bond lengths, together with one oxygen and one nitrogen‐donor atom from the HL^−^ ligand. These polymeric chains are further assembled within the crystal packing through hydrogen bonding, electrostatic interactions, van der Waals forces, and π–π stacking interactions, as described below.

The coordination environment around Hg(II) in the asymmetric unit of compound (**1**) is represented in Figure 1a, while the corresponding coordination polyhedron is illustrated in Figure 1b.

**FIGURE 1:**
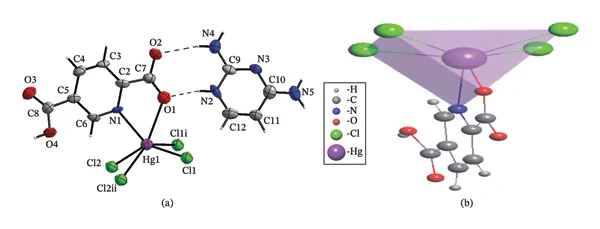
(a) The coordination environment and (b) the coordination polyhedron of the Hg^2+^ in (**1**). Symmetry code: (i) *x* − 1, *y*, *z*; (ii) *x* + 1, *y*, *z*.

The extended one‐dimensional chain architecture arising from the μ‐Cl bridging interactions is presented in Figure 2.

**FIGURE 2:**
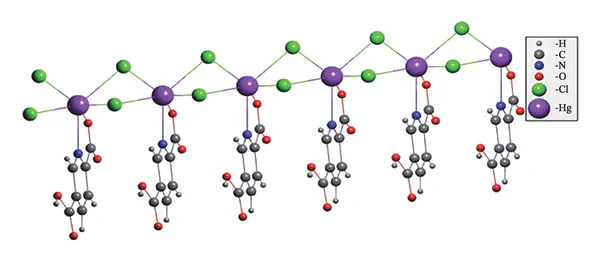
Arrangement of chlorine atoms as bridges in the polymer structure of (**1**).

The protonated 2,4‐diaminopyrimidine does not coordinate to the Hg(II) center but is incorporated into the crystal structure through electrostatic and hydrogen‐bonding interactions. As given in Table S1, the bond lengths are as follows: for oxygen and nitrogen atoms of HL^−^ ligand, Hg1‐*N*1 = 2.364(6) Å and Hg1‐O1 = 2.502(8) Å, and four bridged chlorine atoms from the mercuric chloride salt, Hg1‐Cl1 = 2.356(2), Hg1‐Cl1i = 3.077(2), Hg1‐Cl2 = 2.422(2), and Hg1‐Cl2ii = 3.287(2) Å. Additionally, the angles around the central atom are measured as follows: O1‐Hg1‐Cl1 at 93.2(2)°, O1‐Hg1‐Cl1i at 71.6(2)°, Cl1‐Hg1‐Cl1i at 89.1(2)°, N1‐Hg1‐Cl2ii at 84.9(2)°, Cl2‐Hg1‐Cl2ii at 83.22(6)°, and Cl2‐Hg1‐N1 at 93.2(2)°. These measurements indicate that the coordination geometry of compound (**1**) adopts a slightly distorted triangular prismatic configuration (Figure 1b).

Further structural analysis of compound (**1**) was performed by studying the C–O bond lengths of the carboxylate groups, which provide valuable information on the deprotonation degree and coordination mode of the HL^−^ ligand. In the free ligand, the typical bond lengths of C–O and C=O bonds are 1.307 and 1.205 Å, respectively. In compound (**1**), the following bond lengths were found: 1.198(1) Å for C8–O3; 1.269(1) Å for C8–O4; 1.261(1) Å for C7–O2; and 1.233(1) Å for C7–O1. The great difference between the C8–O3 and C8–O4 bond lengths, together with their close agreement with the typical C=O and C–O bond lengths of the free ligand, indicates that resonance delocalization is not established within this carboxyl group. Consequently, the C8–O3 bond possesses greater double‐bond character, while the C8–O4 bond possesses greater single‐bond character. In contrast, the relatively similar C7–O1 and C7–O2 bond lengths indicate the presence of resonance delocalization within this carboxylate group. The C7–O1 bond length of 1.233(1) Å and the C7–O2 bond length of 1.261(1) Å indicate deprotonation at the O2 atom with transfer of the released proton to the nitrogen atom of the 2,4‐diaminopyrimidine group. Accordingly, proton transfer from the carboxylic acid group to the 2,4‐diaminopyrimidine molecule results in the formation of the anionic coordination polymeric species [Hg(HL) (μ‐Cl)_2_]^−^ and protonated [HL′]^+^ within the crystal structure. The electrostatic attraction between these oppositely charged species, together with hydrogen‐bonding interactions, contributes to the stabilization of the one‐dimensional coordination polymer [36].

The crystal packing of compound (**1**) is stabilized by a combination of supramolecular interactions, including hydrogen bonding, electrostatic interactions, π–π stacking interactions, and van der Waals forces. These noncovalent interactions support the arrangement of the one‐dimensional coordination polymer formed through μ‐Cl bridges and contribute to the stability of the crystal structure [37].

Hydrogen bonding plays an important role in the stabilization of the crystal packing of compound (**1**). The carboxylate oxygen atoms of the deprotonated HL^−^ ligand, together with the amino groups of the protonated [HL′]^+^, provide suitable hydrogen‐bond donor and acceptor sites for establishing an extensive hydrogen‐bonding network. As shown in Figure 3, intermolecular hydrogen bonds are established between the oxygen atoms of the HL^−^ ligand and the hydrogen atoms of adjacent HL^−^ ligands as well as protonated [HL′]^+^ molecules. A hydrogen bond with an H⋯O separation of 1.792 Å is observed between the oxygen atom of one HL^−^ ligand and the hydrogen atom of another HL^−^ ligand. In addition, two hydrogen bonds are formed between the oxygen atoms of the HL^−^ ligand and the hydrogen atoms of the NH_2_ groups of the protonated [HL′]^+^ molecules (H42⋯O2 and H51⋯O3). Another N–H⋯N hydrogen bond is observed between an amino hydrogen atom of one [HL′]^+^ molecule and the ring nitrogen atom of an adjacent [HL′]^+^ molecule (H41⋯N3). An additional N–H⋯O hydrogen bond is formed between the ring NH group of [HL′]^+^ and the oxygen atom of the HL^−^ ligand (H2⋯O1). Furthermore, a weak N–H⋯Cl hydrogen bond is observed between the chloride atom and an NH_2_ hydrogen atoms of the protonated [HL′]^+^ species (H52⋯Cl2). The complete hydrogen‐bond geometry is summarized in Table 4 [38, 39].

**FIGURE 3:**
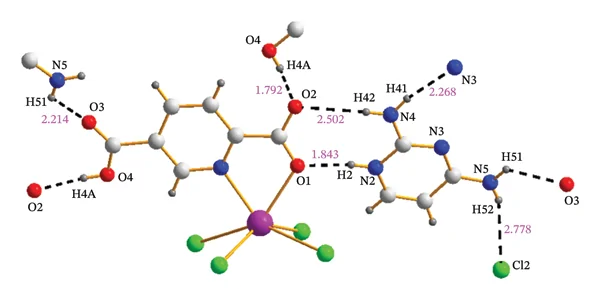
Hydrogen‐bonding interactions in (**1**).

**TABLE 4:** The hydrogen‐bond details, distances (Å), and angles (°) for (**1**) and (**2**).

**(a) crystal (1)**				
***D*—H⋯*A***	***D*—H**	**H⋯*A***	***D*⋯*A***	***D*—H⋯*A***

O4—H4*A*⋯O2^i^	0.82	1.79	2.606 (10)	171
N2—H2⋯O1	0.86	1.84	2.680 (10)	161
N4—H41⋯N3^ii^	0.86	2.27	3.114 (12)	168
N4—H42⋯O2	0.86	2.50	3.306 (11)	158
N5—H51⋯O3^iii^	0.86	2.21	2.833 (12)	129
N5—H52⋯Cl2^iv^	0.86	2.78	3.595 (10)	159

Symmetry codes: (i) *x* + 1, −*y* + 1/2, *z*−1/2; (ii) −*x*, −*y* + 1, −*z*+2; (iii) −*x* + 1, *y* + 1/2, −*z* + 3/2; (iv) −*x* + 1, −*y* + 1, −*z* + 1.				

**(b) crystal (2)**				
***D*—H⋯*A***	***D*—H**	**H⋯*A***	***D*⋯*A***	***D*—H⋯*A***

N2—H2⋯O5^i^	0.86	1.77	2.625 (10)	173
N3—H31⋯O2	0.87	2.29	2.952 (9)	134
N3—H32⋯O5^i^	0.87	2.59	3.329 (9)	144
N4—H41⋯O3^ii^	0.86	2.07	2.793 (8)	141
N4—H42⋯O4^i^	0.86	1.86	2.720 (9)	176

Symmetry codes: (i) *x*, *y* + 1, *z*; (ii) *x*, −*y* + 3/2, *z* + 1/2.				

In addition to hydrogen bonding, electrostatic interactions between the oppositely charged [HL′]^+^ cations and [Hg(HL) (μ‐Cl)_2_]^−^ units also contribute to the stabilization of the crystal packing of compound (**1**). π–π stacking interactions, together with van der Waals forces, also participate in the organization of the crystal packing. As shown in Figure 4a, π–π stacking interactions are observed between the aromatic pyridine and pyrimidine rings within the crystal structure [40]. The centroid‐to‐centroid separation between the interacting aromatic rings is 3.846 Å (Figure 4b), which is within the commonly accepted range of 3–5 Å for π–π stacking interactions [41]. These interactions further support the compact arrangement of the crystal structure.

**FIGURE 4:**
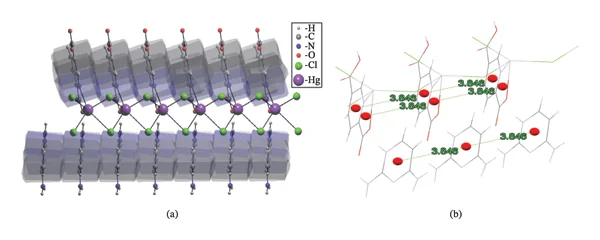
(a) π‐π Interactions and (b) the distance between the aromatic rings and the length of the π‐π interactions in (**1**).

The overall packing arrangement of compound (**1**) is shown in Figure 5. Figure 5a presents the two‐dimensional packing arrangement, whereas Figure 5b illustrates the layered packing arrangement of the crystal structure. Overall, compound (**1**) forms an infinite one‐dimensional coordination polymer through μ‐Cl bridges between adjacent Hg(II) centers.

**FIGURE 5:**
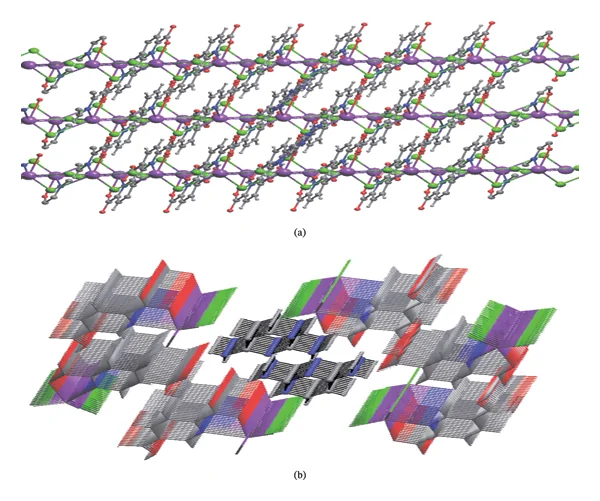
(a) 2D overview and (b) the layered packing arrangement of (**1**).

The polymeric chains are further stabilized within the crystal packing by hydrogen bonding, electrostatic interactions, π–π stacking interactions, and van der Waals forces.

#### 3.2.2. Crystal Structure and Supramolecular Features of Compound (**2**)

SCXRD analysis revealed that compound (**2**) is a mercury(II) coordination compound crystallizing in the monoclinic crystal system with the space group I2/a. The asymmetric unit contains half of the coordination compound, while the complete structure is generated through a crystallographic symmetry plane passing through the central Hg2 atom. The coordination environment for the Hg(II) centers is illustrated in Figure 6a, while the coordination polyhedra for the Hg(II) centers are provided in Figure 6b. Structural analysis of compound (**2**) revealed two crystallographically independent Hg(II) centers, designated Hg1 and Hg2. Application of crystallographic symmetry generates the complete trinuclear coordination unit, which consists of two symmetry‐equivalent four‐coordinate Hg1 centers and one seven‐coordinate Hg2 center.

**FIGURE 6:**
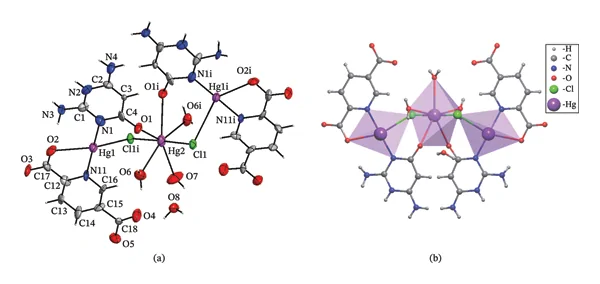
(a) View of the molecular structure of compound (**2**) and (b) the coordination polyhedra around the Hg^2+^ cations. Symmetry code: (i) 1.5‐*x*, *y*, 1 − *z*.

For the first type of coordination site, each four‐coordinate Hg1 center is coordinated by the following ligands: one oxygen atom and one nitrogen atom from the pyridine‐2,5‐dicarboxylate ligand, one nitrogen atom from the 2,4‐diamino‐6‐hydroxypyrimidine ligand, and one bridging chloride ligand. A distorted tetrahedral geometry is observed for the Hg1 ion center. In the case of the second type of coordination sites, the following ligands are present for each seven‐coordinate Hg2 center: two oxygen atoms from two 2,4‐diamino‐6‐hydroxypyrimidine ligands, three oxygen atoms from coordinated water molecules, and two bridging chloride ligands, which result in a distorted pentagonal bipyramidal geometry. In addition, one lattice water molecule is present in the crystal structure.

Bond lengths for the crystallographically independent Hg(II) sites are provided in Table S1. For the four‐coordinate Hg1 center, the bond lengths are as follows: Hg1‐O2 = 2.560(6) Å, Hg1‐*N*1 = 2.046(7) Å, Hg1‐N11 = 2.103(6) Å, and Hg1−Cl1i = 3.258(6) Å. For the seven‐coordinate Hg2 center, the corresponding bond distances are Hg2−O1 = 2.855(5) Å, Hg2‐O1i = 2.855(5) Å, Hg2‐O6 = 2.833(6) Å, Hg2‐O6i = 2.833(6) Å, Hg2‐O7 = 2.810 (6) Å, Hg2‐Cl1 = 2.315(2) Å, and Hg2‐Cl1i = 2.315(2) Å. The approximate equality of the five bond lengths (Hg2‐O1, Hg2‐O1i, Hg2‐O6, Hg2‐O6i, and Hg2‐O7) suggests that they are positioned in the equatorial position, while the equality of the two bond lengths Hg2‐Cl1 and Hg2‐Cl1i indicates that they are situated in the axial position, implying a distorted pentagonal bipyramidal structure.

Furthermore, the angles defining the coordination environment of the central atoms are reported as follows: The angles around the four‐coordinate mercury atom are as follows: N1‐Hg1‐O2 is 108.2(3)°, O2‐Hg1‐N11 is 72.1(2)°, N1‐Hg1‐Cl1i is 97.3(2)°, N11‐Hg1‐Cl1i is 82.9.3(2)°, N1‐Hg1‐N11 is 179.3(3)°, and O2‐Hg1‐Cl1i is 129.9(2)°. Also, the angles around the seven‐coordinate mercury atom are as follows: O6‐Hg2‐O7 at 79,99(13)°, O7‐Hg2‐O6i at 79.99(13)°, O6i‐Hg2‐O1i at 62.6(2)°, O1‐Hg2‐O1i at 75.2(2)°, O1‐Hg2‐O6 at 62.6(2)°, and Cl1‐Hg2‐Cl1i at 177.6(2)°.

Further evaluation of the Hg2 coordination geometry can be obtained from the O–Hg–O bond angles. In an ideal pentagonal bipyramidal geometry, the O–Hg–O angles within the equatorial plane are expected to be close to 72°. In compound (**2**), these angles range from 62.6° to 79.99°, indicating a moderate distortion from the ideal geometry while remaining consistent with a distorted pentagonal bipyramidal environment.

Further structural analysis of compound (**2**) was carried out by examining the C–O bond lengths of the carboxylate groups, which provide useful information about proton transfer and the coordination mode of the ligands. In the free ligand, the typical C–O and C=O bond lengths are approximately 1.30 and 1.20 Å, respectively. In compound (**2**), the C17–O2 and C17–O3 bond lengths are 1.278 and 1.254 Å, respectively, whereas the C18–O4 and C18–O5 bond lengths are 1.203 and 1.245 Å. Compared with the corresponding values of the free ligand, these differences indicate variations in the electron distribution within the two carboxyl groups [42]. The considerable difference in the C18‐O4 and C18‐O5 bond lengths, as well as the similarity of their lengths to the typical lengths of C=O and C‐O bonds, suggests that there is no electron delocalization within this carboxyl group. Therefore, the C18‐O4 bond has greater double‐bond character, while the C18‐O5 bond has greater single‐bond character. On the contrary, the similarity in the lengths of the C17‐O2 and C17‐O3 bonds indicates electron delocalization in this carboxyl group. The comparatively short length of the C17‐O2 bond is connected with the deprotonation of the O2 atom and its coordination to the Hg1 center. Similarity in bond lengths between C17‐O2 and C17‐O3 bonds may be due to delocalization of electrons in the carboxylate ion. These structural features are consistent with proton transfer from pyridine‐2,5‐dicarboxylic acid to the 2,4‐diamino‐6‐hydroxypyrimidine ligand. Because both ligands are directly coordinated to Hg(II) ions, the crystal structure is best described as a neutral coordination compound.

The crystal packing of compound (**2**) is governed by supramolecular interactions, such as hydrogen bonding, π‐π interactions, and van der Waals interactions to develop the three‐dimensional supramolecular structure using the coordination compound. Hydrogen bonding is one of the most important features in the creation of the crystal structure of compound (**2**). The coordinated pyridine‐2,5‐dicarboxylate ligands, the coordinated 2,4‐diamino‐6‐hydroxypyrimidine ligands, and both coordinated and lattice water molecules serve as donors and acceptors for hydrogen bonds that provide an extended hydrogen‐bonding network. It is clear from Figure 7 that three hydrogen bonds are formed between the oxygen atoms of the pyridine‐2,5‐dicarboxylate ligands and the amino hydrogen atoms of the coordinated 2,4‐diamino‐6‐hydroxypyrimidine ligands (H41⋯O3, H42⋯O4, and H32⋯O5). The pyridine‐2,5‐dicarboxylate ligands also form a hydrogen bond with the ring NH group of the 2,4‐diamino‐6‐hydroxypyrimidine ligand (H2···O5). The pyridine‐2,5‐dicarboxylate ligands form another hydrogen bond with the water molecules (H82⋯O2). The 2,4‐diamino‐6‐hydroxypyrimidine ligands form hydrogen bonds with the water molecules (H81⋯O1 and H62⋯O1). There is also a hydrogen bond between the water molecules (H61⋯O8). In addition, a hydrogen bond is observed between the coordinated chloride atom and a water molecule (H62⋯Cl1). The complete geometry of the hydrogen bonds is described in Table 4.

**FIGURE 7:**
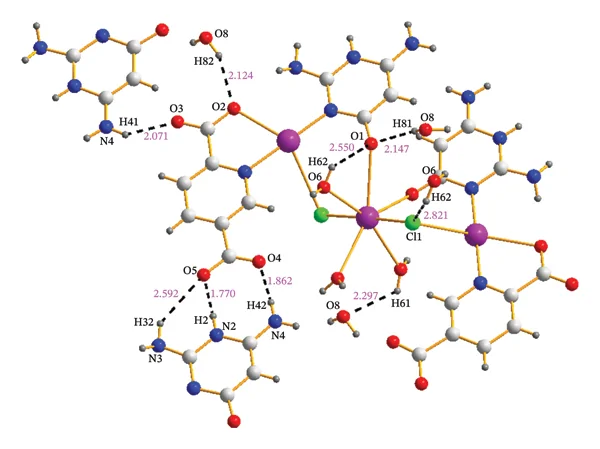
The hydrogen bonds in (**2**).

Proton transfer between pyridine‐2,5‐dicarboxylic acid and 2,4‐diamino‐6‐hydroxypyrimidine generates hydrogen‐bond donor and acceptor sites, thereby facilitating the formation of an extended hydrogen‐bonding network throughout the crystal structure. However, apart from hydrogen bonding, other interaction forces also contribute to stabilizing the crystal lattice, namely π–π stacking interactions and van der Waals forces. Figure 8a shows the π–π stacking interaction between the aromatic rings of the pyridine and pyrimidine ligands [41]. The centroid‐to‐centroid distance between the interacting aromatic rings is 3.417 Å (Figure 8b). This distance falls within the 3–5 Å range typical for π–π stacking interactions [42]. Such interactions further contribute to the compact crystal packing.

**FIGURE 8:**
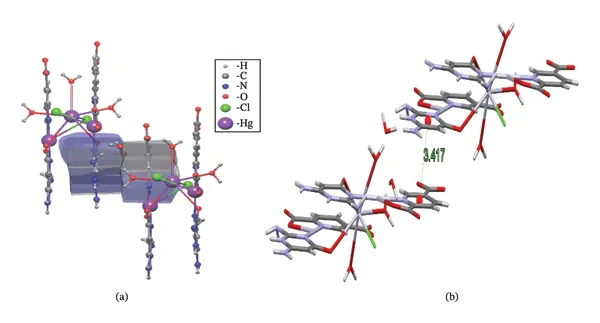
(a) π‐π Interactions and (b) the distance between the aromatic rings and the length of the π‐π interactions in (**2**).

The overall packing arrangement of compound **(2)** is illustrated in Figure 9. The crystal packing is maintained by hydrogen bonding, π–π stacking interactions, and van der Waals forces.

**FIGURE 9:**
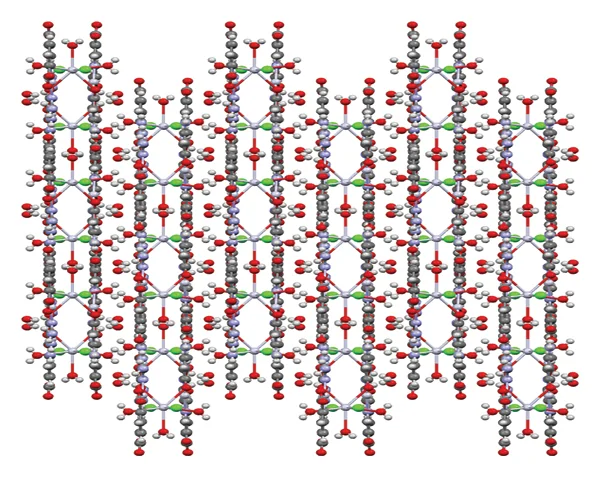
An overview of (**2**).

#### 3.2.3. Hirshfeld Surface Analysis (HSA)

The intermolecular interactions and crystalline structures of two coordination compounds were extensively examined using HSA and two‐dimensional fingerprint plots generated with CrystalExplorer 3.0 software [30, 31]. The HSA technique offers a novel approach to elucidating and quantifying the complexities and magnitudes of intermolecular interactions present in molecular crystals. It delineates the theoretical boundary between a given molecule and its surroundings in the crystal environment, encompassing all other molecular entities and any void space. A partitioning of the electron density of the crystal into molecular contributions according to the Stockholder scheme enables the construction of the surface. The surfaces can be represented with properties indicating the type and strength of intermolecular interactions, including *d*_*i*_ for the distance to the nearest nucleus inside and de for the nearest nucleus outside the surface. Two‐dimensional fingerprint plots are derived from the Hirshfeld surface by plotting *d*_*i*_ versus *d*_*e*_ for each surface point, providing a quantitative description of intermolecular contacts, whereas the *d*_norm_ surface offers a qualitative visualization of the strength and proximity of these interactions; these plots provide insight into different kinds of contacts with relative contributions to the Hirshfeld surface, including hydrogen bonding, van der Waals interactions, and π–π stacking.

A representative Hirshfeld surface illustrating the various intermolecular contacts present in compounds (**1**) and (**2**) is shown in Figure 10.

**FIGURE 10:**
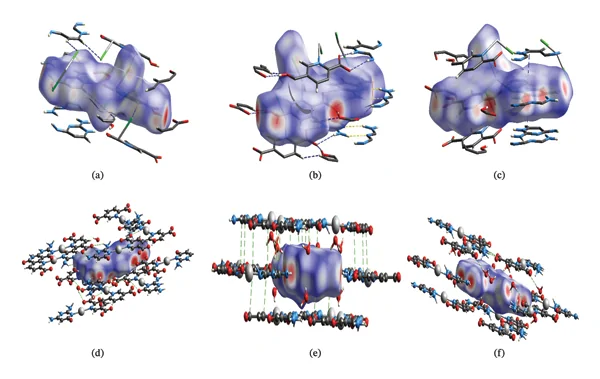
(a) Symmetric Cl⋯H contacts mapped on the Hirshfeld surface of compound (**1**). (b) Close O⋯H and N⋯H contacts visualized on the Hirshfeld surface of compound (**1**). (c) Long‐range C⋯C contacts represented on the Hirshfeld surface of compound (**1**). (d) Short O⋯H contacts highlighted on the Hirshfeld surface of compound (**2**). (e) Long‐range C⋯C contacts visualized on the Hirshfeld surface of compound (**2**). (f) Symmetric C⋯H contacts mapped onto the Hirshfeld surface of compound (**2**).

In coordination compound (**1**), the dominant intermolecular contacts are H⋯H, O⋯H, C⋯O, and Cl⋯H interactions, indicating the combined influence of van der Waals forces and hydrogen bonding in stabilizing the crystal lattice. In contrast, the HSA of coordination compound (**2**) reveals that the principal contributions arise from O⋯H, H⋯H, C⋯H, and C⋯C contacts, suggesting that the crystal packing is primarily governed by hydrogen bonding, dispersive interactions, and weak π–π or C⋯C contacts. The lattice structures of both complexes are therefore stabilized by a combination of hydrogen bonds and dispersive forces, albeit with distinct interaction profiles. The major contribution of O⋯H contacts for all of the compounds is due to the presence of carboxylic acid functional groups and some water molecules involved in the molecular framework, which leads to the formation of strong hydrogen bonds among themselves and with neighboring molecules.

The respective 2D fingerprint plots of compounds (**1**) and (**2**) provide a quantitative depiction of the different intermolecular contacts, allowing the similarities and differences in crystal packing to be clearly visualized (Figure 11).

FIGURE 11(a) 2D Fingerprint plots of major contacts in (**1**) and (b) (**2**).

**Figure:**
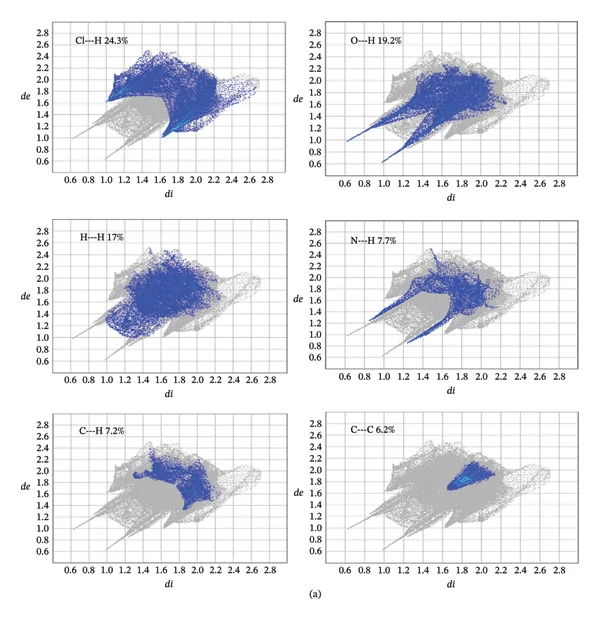

**Figure:**
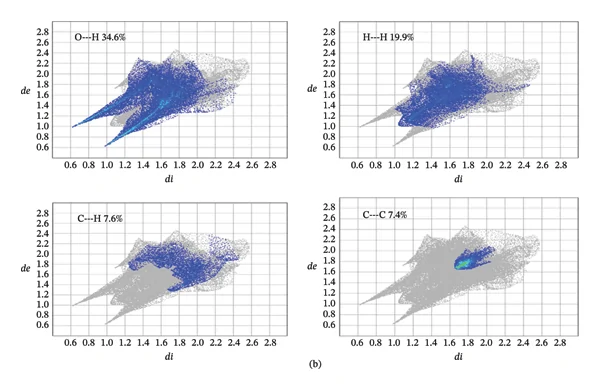

The bar charts summarizing the contributions of various intermolecular interactions in coordination compounds (**1**) and (**2**) illustrate the relative percentages of each contact type, offering a clear comparison of interaction profiles between the two compounds (Figure 12).

**FIGURE 12:**
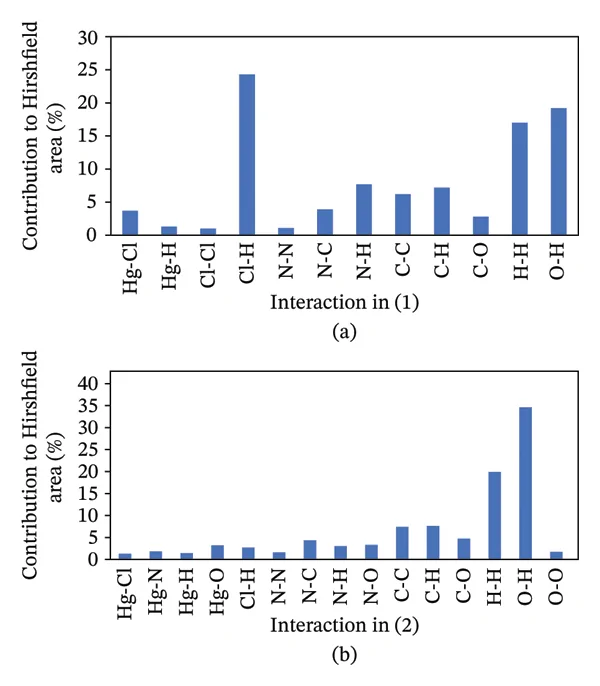
The relative contribution of intermolecular forces to the Hirshfeld surface area for (a) (**1**) and (b) (**2**).

Analysis indicates that both compounds are compact and planar in geometry and show strong hydrogen bonding with minimal van der Waals interaction with the external environment. Furthermore, analysis shows that the primary difference between the two compounds lies in the fact that additional hydrogen bonding is afforded by the presence of the pyrimidine ligand in (**1**) and (**2**), as well as a structural alteration to the molecule and, hence, the void space with which it interacts.

### 3.3. Characterization of Compounds (1′) and (2ʹ)

#### 3.3.1. NMR

To identify the synthesized powder sample of coordination compounds, the ^1^H NMR spectra of compounds (**1′**) and (**2′**) were acquired in deuterated dimethylsulfoxide solvent. The ^1^H NMR spectrum of compound (**1′**) is shown in Figure S1a. According to Figure S1a, the acidic hydrogen **A** appears as a broad peak at 13.20 ppm, and hydrogen **B** appears as a doublet at 9.18 ppm with a coupling constant of 2.02 Hz. Hydrogen **C** appears as a doublet of doublets in the region of 8.52 ppm with coupling constants of 8.52 and 2.02 Hz, and hydrogen **D** appears as a doublet at 8.32 ppm with a coupling constant of 8.52 Hz. Hydrogen **E** appears as a singlet at 7.95 ppm, and hydrogen **F** appears as a somewhat broader singlet at 7.88 ppm. Hydrogen **G** appears as a doublet at 7.73 ppm with a coupling constant of 7.01 Hz, hydrogen **H** appears as a sharp peak at 7.63 ppm, and hydrogen **I** also appears as a doublet at 6.00 ppm with a coupling constant of 7.01 Hz [43–45]. As shown in Figure S1b, eleven carbon peaks are observed. Carbon **J** is observed at 97.41 ppm, carbon **B** at 125.31 ppm, carbon **C** at 131.64 ppm, carbon **D** at 140.56 ppm, carbon **E** at 143.72 ppm, carbon **K** at 149.99 ppm, carbon **A** at 152.67 ppm, carbon **I** at 156.65 ppm, carbon **F** and **G** at 165.36 ppm, and carbon **H** at 166.78 ppm [9].

Figure S2 shows the ^1^H NMR spectrum of compound (**2′**). Hydrogen **A** appears as a singlet at 9.92 ppm, hydrogen **B** is a doublet at 9 ppm (*J* = 2.06 Hz), hydrogen **C** is a doublet of doublets around 8.45 ppm (*J* = 8.10 and 2.06 Hz), and hydrogen **D** is a doublet at 8.22 ppm (*J* = 8.10 Hz). Hydrogens **E** and **F** show two singlets at 6.15 and 5.94 ppm, respectively, while another singlet for hydrogen **G** appears at 4.46 ppm [44, 46].

#### 3.3.2. IR Spectra

The FTIR spectra of the free ligands and sonochemically prepared samples (**1′**) and (**2′**) are presented in Figure S3. FTIR spectroscopy was employed to investigate the characteristic vibrational bands of the ligands and to evaluate the changes associated with Hg(II) coordination. In the FTIR spectrum of H_2_L, the broad absorption band related to the O–H stretching vibration was observed at 3457 cm^−1^. For samples (**1′**) and (**2′**), broad absorptions appeared in the ranges of 2400–3500 and 2500–3500 cm^−1^, respectively, which can be attributed to O–H stretching vibrations associated with hydrogen‐bonded groups and water molecules present in the structures. The stretching vibrations associated with the NH_2_ groups of L′ and HL″ were observed in the range of 3382–3473 cm^−1^, while the corresponding bands in (**1′**) and (**2′**) appeared within the range of 3330–3538 cm^−1^, indicating changes in the local chemical environment of these groups after complex formation.

The C–H stretching vibrations of H_2_L, L′, and HL″ were detected in the range of 2946–3014 cm^−1^, whereas the corresponding bands in (**1′**) and (**2′**) appeared within the range of 2920–3116 cm^−1^. The absorption bands associated with the carboxylic groups of H_2_L were observed at 1735 and 1535 cm^−1^. After coordination with Hg(II), variations were observed in the carboxyl‐containing region. In the spectra of (**1′**) and (**2′**), the asymmetric stretching vibrations of coordinated and noncoordinated carboxylate groups appeared in the ranges of 1580–1625 and 1695–1714 cm^−1^, respectively. The corresponding symmetric stretching vibrations of carboxylate groups were observed in the ranges of 1390–1436 and around 1480 cm^−1^. These changes indicate modification of the electronic environment of the carboxylate groups upon interaction with Hg(II) centers.

The absorption bands observed at 1589 and 1369 cm^−1^ in the FTIR spectrum of H_2_L were assigned to C=N and C=C stretching vibrations of the pyridine ring, respectively. In the spectra of (**1′**) and (**2′**), the corresponding pyridine ring vibrations appeared in the range of 1349–1635 cm^−1^. Furthermore, the bands around 1216 cm^−1^ in H_2_L and within the range of 1039–1280 cm^−1^ in (**1′**) and (**2′**) were attributed to C–N and C–C stretching vibrations of the pyridine ring. The weak absorption bands located in the range of 447–528 cm^−1^ were assigned to Hg–O and Hg–N stretching vibrations, providing further evidence for metal–ligand coordination.

The separation values between the asymmetric and symmetric stretching frequencies of the carboxylate groups (Δ*ν* = $\nu_{\text{as}\left({\text{COO}}^{-}\right)}-\nu_{\text{s}\left({\text{COO}}^{-}\right)}$) for samples (**1′**) and (**2′**) were calculated as 214, 235, and 146 cm^−1^. Compared with the corresponding value of the free ligand (approximately 200 cm^−1^), these variations indicate changes in the coordination environment of the carboxylate groups after complex formation. The observed differences are consistent with the coordination modes of the ligands determined by SCXRD analysis [9, 10, 47, 48].

#### 3.3.3. PXRD Spectral Studies

The PXRD patterns of the powder samples of coordination compounds **(1′)** and (**2′**) were compared with the corresponding simulated patterns derived from the SCXRD data of compounds (**1**) and (**2**), respectively (see Figure S4). The experimental PXRD patterns exhibited good agreement with the corresponding simulated patterns, particularly with respect to the positions of the principal diffraction reflections. A quantitative comparison of the characteristic diffraction peaks was performed by comparing the experimental and simulated 2*θ* values. For compound (**1**)/(**1′**), 14 characteristic reflections were compared, giving a mean absolute difference in 2*θ* of approximately 0.033°, with a standard deviation of 0.036°. For compound (**2**)/(**2′**), 20 characteristic reflections were compared, giving a mean absolute difference in 2*θ* of approximately 0.028°, with a standard deviation of 0.025°. These small differences in peak positions support a high degree of correspondence between the experimental PXRD patterns of the sonochemically prepared powders and the patterns calculated from the corresponding single‐crystal structures.

Minor variations in the relative intensities of some reflections were observed between the experimental and simulated patterns. Such differences are reasonably attributed to preferred orientation effects inherent to powder measurements, as well as differences in crystallite size, particle morphology, aggregation, and sample preparation. Importantly, the characteristic diffraction features predicted from the single‐crystal structures are reproduced in the experimental PXRD patterns of both (**1′**) and (**2′**), and no additional intense reflections attributable to a major secondary crystalline phase were detected within the sensitivity of the PXRD measurements. These observations support the conclusion that the sonochemically prepared samples (**1′**) and (**2′**) correspond to the same predominant crystalline phases as compounds (**1**) and (**2**), respectively. It should be noted that PXRD alone cannot establish complete chemical compositional identity or exclude minor amorphous components or trace impurities below the detection limit; therefore, the chemical identities of the compounds are supported by the single‐crystal structural determinations and the complementary characterization data presented in this work.

The Bravais–Friedel–Donnay–Harker (BFDH) method was employed to predict the theoretical crystal morphology of both compounds based on their internal crystal structures. For compound **(1)**, BFDH‐based morphology simulations suggest an elongated prismatic crystal habit enclosed predominantly by the (020), (011), and (002) crystal faces.

The predicted morphology suggests that the crystals preferentially grow along the b‐axis. For consistency, the progressive inclusion of Miller‐indexed planes with increasing diffraction intensity is likewise illustrated for compound (**1**) in Figure S5.

The PXRD pattern of compound (**2**) exhibits reflections corresponding to the (011), (110), (200), (20‐2), and (112) crystallographic planes, which were subsequently considered in the BFDH morphology analysis. In Figure S5, all the Miller indices of the crystal faces are included with increasing intensity, highlighting the relationship between crystal facets and their overall shape.

These observations suggest that several crystal facets contribute to crystal growth, whereas the low‐energy planes predominantly determine the external morphology, giving rise to an elongated prismatic crystal habit. Morphology simulations further indicate that the low‐index (1−10) facet is associated with the preferred growth direction (PGD), while the remaining facets contribute to the development of the overall crystal habit. Crystal growth is likewise preferentially directed along the b‐axis. Overall, the consistency among PXRD phase analysis, morphology simulations, and FESEM observations provides a coherent and qualitative description of the crystal growth behavior of both compounds, without the need for additional crystallographic assumptions.

#### 3.3.4. FESEM and EDS Analysis

The scanning electron micrographs of compounds (**1′**) and (**2′**) are shown in Figure 13. FESEM analysis shows that compound (**1′**) forms plate‐like microcrystals with micrometer‐scale lateral dimensions and a nanoscale thickness of ∼80 ± 20 nm. The particles display an elongated plate‐like morphology, which is in reasonable agreement with the elongated prismatic crystal habit predicted by the BFDH model. Compound (**2′**) consists of elongated plate‐like particles with a faceted morphology and submicrometer lateral dimensions (∼300–500 nm), with a minimum thickness of ∼90 ± 20 nm.

**FIGURE 13:**
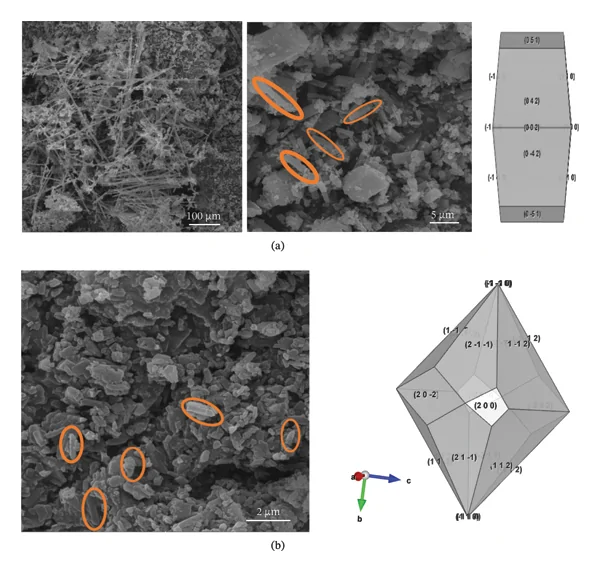
Comparison of FESEM images and BFDH‐predicted morphologies for (a) compound (**1′**) and (b) compound (**2′**), showing similarities in crystal habit and overall morphological features.

The faceted anisotropic morphology observed in FESEM images is in reasonable agreement with the elongated prismatic crystal habit predicted by the BFDH model. The average particle thickness of compounds (**1′**) and (**2′**) was measured using ImageJ, and the distribution is shown in Figure 14.

**FIGURE 14:**
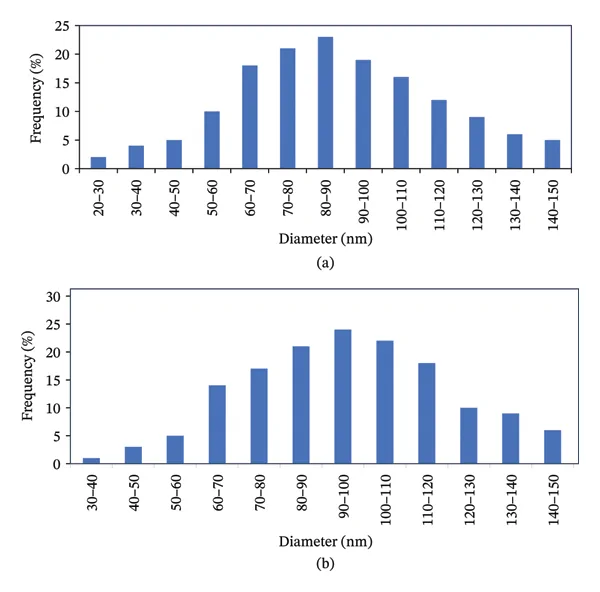
Histogram showing the average particle thickness distribution of compounds (a) (**1′**) and (b) (**2′**) measured using ImageJ.

Energy‐dispersive X‐ray spectroscopy (EDS) was used to determine the elemental composition of both compounds to confirm the structure‐based observations made previously (see Figures S6a and S6b). EDS analysis of both compounds confirmed that they contained mercury, oxygen, nitrogen, chlorine, and carbon, consistent with their proposed chemical structures. The close match between the experimentally determined elemental percentages and the theoretically calculated values provides confidence in the elemental compositions. Furthermore, these results provide additional support for the structural characterization discussed above.

#### 3.3.5. Dispersion Behavior in Aqueous Suspension: DLS and Zeta Potential Analysis

To further examine the dispersion behavior of the coordination compounds in aqueous suspension, DLS and zeta potential measurements were performed (Figure S7). Compound (**1**) exhibited an average hydrodynamic diameter (*D*_mean_) of 908.9 nm with a polydispersity index (PDI) of 0.15477. Under the same conditions, compound (**2**) showed a *D*_mean_ of 1058.1 nm together with an exceptionally low PDI value of 0.00010, indicating a highly uniform aggregate size distribution.

The hydrodynamic diameters obtained from DLS fall within the submicrometer‐to‐micrometer range, whereas FESEM observations reveal anisotropic crystalline particles with nanoscale thickness. This difference originates from the distinct principles of the two techniques. FESEM characterizes individual crystalline particles after drying, while DLS measures the hydrodynamic diameter of particles dispersed in solution, where the measured size may also include particle aggregates and the surrounding solvation layer. The DLS results are consistent with the zeta potential measurements. Mean zeta potentials of −0.42 mV for compound (**1**) and −1.21 mV for compound (**2**) indicate poor colloidal stability and a pronounced tendency for aggregation in aqueous suspension. Although compound (**2**) exhibited a slightly larger hydrodynamic diameter than compound (**1**), its extremely low PDI (0.00010) compared with that of compound (**1**) (0.15477) suggests a much narrower aggregate size distribution. Therefore, the particle sizes determined by DLS should be regarded as the hydrodynamic dimensions of particle aggregates present in suspension rather than the actual dimensions of the individual crystalline particles observed by FESEM. It is acknowledged that the DLS and zeta potential measurements reported above were performed in general aqueous suspension and not directly within Mueller–Hinton broth, the medium used for the antibacterial disk diffusion and microdilution assays. Directly assessing the stability, aggregation state, and potential Hg^2+^ release of compounds (1′) and (2′) within this biological medium (e.g., via PXRD or UV–Vis monitoring of the suspension after 24‐h incubation) would further strengthen the interpretation of the antibacterial results in terms of the molecular identity of the active species and is proposed as an important direction for future work.

#### 3.3.6. Thermogravimetric Analysis (TGA)

TGA was performed to evaluate the thermal stability of the compounds. Figure S8, which represents the thermal behavior of (**1′**) and (**2′**), can be found in the Supporting Information. The TGA curves exhibited distinct weight‐loss steps corresponding to specific temperature intervals. As observed in Figure S8, compound (**1′**) remains thermally stable up to approximately 250°C. A major weight‐loss step occurs between 226°C and 400°C. During this stage, the compound loses 84.19% of its weight (calculated value: 84.14%). In effect, a sharp and major mass loss corresponding to the decomposition of almost the entire organic framework occurs, leaving a small solid residue at higher temperatures. Finally, the remaining carbonaceous residue is gradually eliminated upon further heating [22].

Compound (**2′**) shows stability until 71°C. The TGA curve exhibits three temperature peaks in the range of 71°C–130°C, 228°C–488°C, and 488°C–800°C, corresponding to the loss of four coordinated and uncoordinated water molecules (Exp. = 5.83%, Calc. = 6.69%), decomposition of the coordinated organic ligands together with mercury‐containing species (Exp. = 73.68%, Calc. = 73.68%), and Hg_2_Cl_2_ component (Exp. = 20.49%, Calc. = 19.62%), respectively. The remaining residue can be assigned to Hg_2_Cl_2_, which decomposes upon further heating up to 800°C, leaving no detectable solid residue at the end of the analysis [21].

#### 3.3.7. Surface Area and Porosity (BET Analysis)

The N_2_ adsorption–desorption isotherms for compound (**1′**) and compound (**2′**) samples are shown in Figure 15a,b, respectively. Both samples show type IV isotherm behavior with H3‐type hysteresis loops, which are found in the 0.40–1.00 *P*/*P*_0_ pressure range, indicating the presence of slit‐shaped mesopores based on IUPAC (2015) classification. The BET surface area for (**1′**) is 9.16 m^2^g^−1^, while that for (**2′**) is 10.16 m^2^g^−1^. The Langmuir surface area for both samples is slightly higher than that obtained from the BET method; however, the BET method yields values for accessible surface areas that are more accurate than those obtained with the Langmuir method.

**FIGURE 15:**
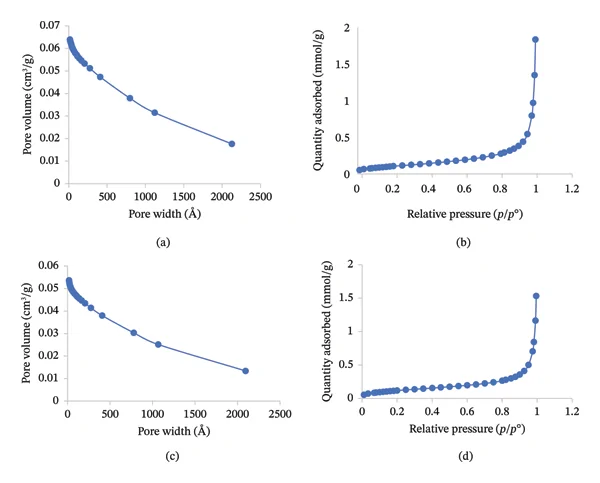
Nitrogen adsorption–desorption analysis for compounds (**1′**) and (**2′**) at 77 K. (a) Corresponding pore volume and pore size distribution derived from the desorption branch for compound (**1′**), (b) adsorption–desorption isotherm of compound (**1′**), (c) corresponding pore volume and pore size distribution derived from the desorption branch for compound (**2′**), and (d) adsorption–desorption isotherm of compound (**2′**).

The BJH pore diameter method was used to calculate the average pore diameter for compound (**1′**) and compound (**2′**), based on the adsorption branches of the adsorption/desorption isotherm data and the pore size distribution curves presented in Figure 15c,d. Based on the average pore diameters of (**1′**) (approx. 12.0 nm) and (**2′**) (approx. 9.6 nm), both compounds can be classified as mesoporous materials according to the IUPAC classification. The measured total pore volume of (**1′**) is 0.064 cm^3^g^−1^; that of (**2′**) is 0.054 cm^3^g^−1^. The analysis has shown that (**2′**) has a slightly higher surface area than (**1′**), but the average pore diameter of (**2′**) is smaller than (**1′**); these structural characteristics may be relevant for adsorption‐related applications [49].

### 3.4. Theoretical Morphology Prediction

According to the BFDH model, crystal faces with larger interplanar spacings (d_hkl_) grow more slowly and therefore dominate the external morphology. These planes can be defined by their Miller indices (also known as hkl), which also provide insight into the ultimate growth of the crystal.

Using BFDH analysis of compound (**1**) (which is a triclinic, P¯1 crystal), the dominant slow‐growing family of faces was found to be as follows: (020), (011), (01‐1), and (002). They represent the highest d_hkl_ values (where *d*_020_ = 15.18 Å and *d*_011_ = 11.88 Å) and thus dominate the external crystal morphology. The faster‐growing planes, which give rise to PGDs, include planes, such as (100), (110), and (1‐10), thereby defining the preferred crystal growth directions. Compound (**1**) exhibits an elongated prismatic crystal habit and primarily grows along the b‐axis. Figure S9a, illustrating the BFDH‐predicted crystal morphology and the calculated dominant crystal faces for compound (**1**), can be found in the Supporting Figures. BFDH analysis of compound (**2**) yielded information similar to that obtained for compound (**1**). Its slow‐growing planes include planes, such as (011), (110), (1‐10), (20‐2), and (112), which correspond well with the PXRD reflections observed at (011), (110), (20−2), and (112). Faster‐growing planes, including (20‐2), (‐202), (200), and (‐200), serve as PGDs. The asymmetric coordination environment around Hg(II), together with the supramolecular interactions present in the crystal structure, is consistent with the formation of slightly thinner crystals with an elongated prismatic crystal habit compared with compound (**1**). The BFDH‐predicted crystal morphology and the corresponding dominant crystal faces of compound (**2**) are illustrated in Figure S9b.

#### 3.4.1. Correlation With PXRD and FESEM

The theoretical BFDH predictions for both compounds show reasonable agreement with the experimental PXRD and FESEM data. The PXRD reflections corresponding to the predicted slow‐growing planes (020), (011), and (002) are consistent with the BFDH prediction for this compound. Further support for the predicted crystal habit is provided by scanning electron microscopic imaging (FESEM), which reveals the predicted elongated plate‐like morphology of the crystals.

For compound (**2**), the PXRD reflections (011), (110), (20‐2), and (112) correspond to the predicted slow‐growing faces, and the FESEM micrographs show that the crystals exhibit a thin, elongated plate‐like morphology, indicating that the majority of the crystal edges are aligned with the predicted growth directions. The comparison of these data suggests that the thicker, elongated plate‐like morphology for compound (**1**) is produced as a result of the symmetrical coordination of Hg(II) in this compound, whereas the thin, elongated prismatic plates of compound (**2**) are influenced by the asymmetrical coordination of Hg(II) in this compound. Overall, it can be concluded that a combination of interplanar spacing (d_hkl_), low‐energy planes, hydrogen bonding, and π–π stacking collectively contributes to the anisotropic growth and external morphology of both compounds. Figure S10 shows the dominant slow‐growing crystal faces identified from the BFDH analysis.

It should be noted that crystal faces exhibiting large relative surface areas in the BFDH morphology predictions for compounds (**1**) and (**2**) are not necessarily associated with the most intense PXRD reflections or the most visually dominant facets in the final morphology representations. The BFDH model correlates crystal growth behavior primarily with interplanar spacings (d_hkl_), from which the relative surface areas of the exposed faces are derived, whereas PXRD peak intensities are governed by structure factors, multiplicity, and preferred orientation effects. Therefore, partial discrepancies between dominant BFDH‐predicted faces and PXRD peak intensities are expected and do not indicate inconsistencies in the structural or morphological interpretations.

### 3.5. Biological Studies

#### 3.5.1. Antibacterial Assay

The antibacterial activity of Hg(II)‐based coordination compounds (**1′**) and (**2′**) was evaluated against sixteen Gram‐positive and Gram‐negative bacterial strains using the disk diffusion and broth microdilution methods.

##### 3.5.1.1. The Disk Diffusion Assay

Overall, compound (**2′**) exhibited greater antibacterial activity than compound (**1′**) against most of the bacterial strains examined. For the Gram‐positive bacterium *Staphylococcus aureus* (ATCC 6538), the measured zone of inhibition was 26.5 and 35.5 mm for compounds (**1′**) and (**2′**), respectively, while penicillin showed a zone of 40 mm (Figure 16). Methicillin‐resistant *Staphylococcus aureus* (MRSA) displayed greater susceptibility to compound (**2′**), exhibiting an inhibition zone of 27 mm compared with 17 mm for compound (**1′**), whereas penicillin showed no detectable antibacterial activity. For *Staphylococcus epidermidis* (ATCC 14990), inhibition zones of 20.5, 26, and 42 mm were observed for compounds (**1′**), (**2′**), and penicillin, respectively. The inhibition zones of Gram‐positive species, including *Streptococcus agalactiae* and *Streptococcus pyogenes*, exhibited relatively low susceptibility to both compounds (**1′**) and (**2′**) (9.5–11 mm). Among the Gram‐negative isolates, *Shigella flexneri*, *Klebsiella pneumoniae*, and *Acinetobacter baumannii* exhibited the greatest susceptibility to compound (**2′**), with inhibition zones of 32.5, 30.5, and 29 mm, respectively. In several cases, these values approached those obtained for the ciprofloxacin control (35–40 mm).

**FIGURE 16:**
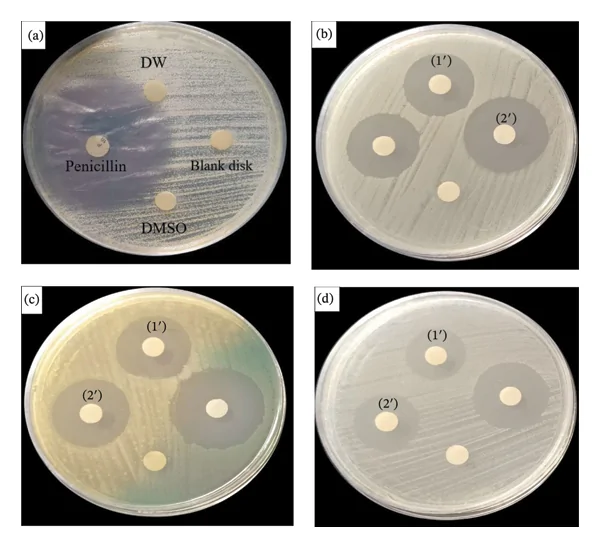
Antibacterial activity assay by the disk diffusion method. (a) The inhibition zone produced by *Staphylococcus aureus* ATCC 6538 when exposed to the control group, (b) the inhibition zone observed for *Escherichia coli* ATCC 8739 treated with compounds (**1′**) and (**2′**) (5 mg/mL). (c) The inhibition zone generated by *Pseudomonas aeruginosa* clinical (PAO1) isolate upon treatment with compounds (**1′**) and (**2′**) (5 mg/mL). (d) The antibacterial effect demonstrated by *Staphylococcus epidermidis* ATCC 14990 in response to compounds (**1′**) and (**2′**) (5 mg/mL).

Figure 17 displays the average diameters of inhibition zones (in mm) recorded in disk diffusion tests for two synthesized mercury coordination compounds ((**1′**) and (**2′**)) against various Gram‐positive and Gram‐negative bacterial strains. The bars on the right represent control antibiotics: penicillin (10 μg) for Gram‐positive strains and ciprofloxacin (5 μg) for Gram‐negative strains. Compound (**2′**) consistently showed larger inhibition zones than compound (**1′**), suggesting superior antimicrobial effectiveness.

**FIGURE 17:**
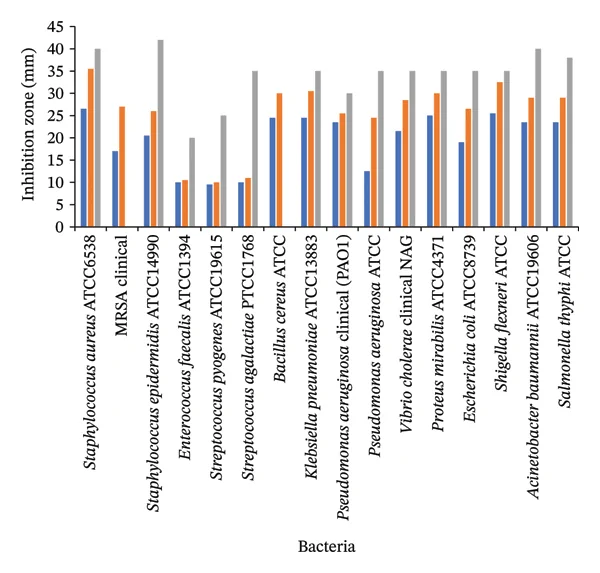
Antimicrobial effectiveness of compound (**1′**) (blue) and compound (**2′**) (orange) in comparison with standard antibiotics (gray).

##### 3.5.1.2. The Broth Microdilution Assay

Quantitative MIC and MBC measurements further confirmed the antibacterial trends observed in the disk diffusion assay (Table 3). Compound (**2′**) demonstrated substantially lower MIC and MBC values than compound (**1′**) for nearly all bacterial strains.

Compound (**2′**) displayed low MICs for most strains (0.12–0.97 μg/mL), while compound (**1′**) needed higher concentrations (0.48–9.76 μg/mL). The greatest sensitivity to compound (**2′**) was recorded in *S. pyogenes* and *S. agalactiae*, both showing an MIC of 0.12 μg/mL and an MBC of 0.24 μg/mL. In the case of compound (**1′**), *S. pyogenes* exhibited the highest susceptibility with both MIC and MBC at 0.12 μg/mL. These findings emphasize the robust overall performance of compound (**2′**) and the particularly strong effect of compound (**1′**) against *S. pyogenes*. For *S. epidermidis*, compound (**2′**) resulted in an MIC/MBC of 0.48 μg/mL, compared to 2.44 μg/mL for compound (**1′**), indicating substantially enhanced antibacterial potency. Concerning the Gram‐negative strains, *Shigella flexneri*, *Salmonella typhi*, and *Vibrio cholerae* showed responses to compound (**2′**) with MICs as low as 0.48 μg/mL. The highest MIC recorded was 4.88 μg/mL for *Pseudomonas aeruginosa*, which still indicated a more favorable profile than compound (**1′**) (9.76 μg/mL). The relatively low MBC values suggest that both compounds may exert bactericidal effects.

Antimicrobial efficacy of coordination compounds (**1′**) and (**2′**) was evaluated using the disk diffusion and broth microdilution techniques. The inhibition zone diameters (mm) obtained from the disk diffusion method, as well as the MIC (μg/mL) and MBC (μg/mL) from the broth microdilution test, are presented in Table 3. Compound (**2′**) demonstrated enhanced antimicrobial effectiveness against most of the strains tested, including multidrug‐resistant organisms, such as MRSA and *A. baumannii*. The data shown represent the averages of two technical replicate measurements obtained within a single assay.

The present study demonstrates the antimicrobial activity of the synthesized Hg(II) coordination compounds (**1′**) and (**2′**) against a broad spectrum of Gram‐positive and Gram‐negative bacteria.

Numerous studies have reported that mercury‐based complexes exhibit potent antimicrobial properties against a broad spectrum of bacterial strains, thereby enhancing their potential as antimicrobial agents [7, 50–53]. The observations imply that the incorporation of mercury‐based complexes into future antimicrobial development may offer a viable alternative in addressing resistant bacterial infections, thus necessitating further exploration of their mechanisms and long‐term safety profiles [7, 54, 55].

Modifications to metal‐ligand frameworks have demonstrated the capacity to enhance antimicrobial effectiveness by entering cells and aiding their movement across the cell wall of multidrug‐resistant *S. aureus* [56], thereby underscoring the pivotal role of ligand design. Irzoqi further accentuates the potential of mercury‐based coordination compounds as potent agents against antibiotic‐resistant bacterial strains [57]. These conclusions are consistent with our findings, which reveal the strong antibacterial activity of Hg(II) complexes, particularly against resistant strains, such as *S. aureus* and *S. epidermidis*.

Despite the encouraging antimicrobial results, the clinical application of mercury‐based agents necessitates a comprehensive evaluation of cytotoxicity and environmental ramifications. Subsequently, the integration of mercury into stable coordination complexes may reduce systemic toxicity while retaining its antimicrobial properties [51, 58, 59]. Mercury ions have the capacity to interfere with bacterial metabolism through their binding to thiol (‐SH) groups present on critical enzymes [60]. The employment of bidentate or multidentate organic ligands, as observed for compounds (**1′**) and (**2′**), serves to diminish ion lability, thereby facilitating a more precise and controlled antibacterial action [57, 61–63].

The current investigation demonstrates the remarkable antibacterial potency of (**2′**) against a broad array of both Gram‐positive and Gram‐negative bacteria, encompassing multidrug‐resistant bacteria, such as MRSA and *A. baumannii*. The MIC of (**2′**) against MRSA was recorded at 2.44 μg/mL, which indicates promising antibacterial activity for a Hg(II) coordination compound [64, 65].

This observation aligns with prior studies that document the antibacterial potency of Hg(II) complexes, particularly against resistant pathogens, such as *S. aureus* and *A. baumannii* [66, 67]. Furthermore, its efficacy against *A. baumannii* (MIC = 0.48 μg/mL) is comparable to that of previously reported metal‐based antimicrobial agents, including silver‐ and gold‐containing materials, which typically require higher concentrations to inhibit bacterial growth [68–71].

Additionally, the relative efficacy of (**2′**) in comparison with traditional antibiotics warrants attention. While ciprofloxacin and penicillin retain efficacy against susceptible strains, (**2′**) exhibited inhibition zones approached those of the standard antibiotics for significant pathogens, including *Shigella flexneri* and *S. aureus*.

As detailed in Section 3.2, compound (**1**) forms an extended one‐dimensional coordination polymer stabilized by μ‐Cl bridges and a compact hydrogen‐bonding network involving the protonated [HL′]^+^ cation (Section 3.2.1, Table 4a), whereas compound (**2**) adopts a discrete trinuclear supramolecular architecture in which the additional hydroxyl group of the 2,4‐diamino‐6‐hydroxypyrimidine ligand and coordinated/lattice water molecules generate a more extensive hydrogen‐bonding network (Section 3.2.2, Figure 7, Table 4b). These structural distinctions in dimensionality, hydration, and hydrogen‐bonding capacity are directly relevant to the differential antibacterial performance discussed below.

Nevertheless, an important question arising from the present findings is why compound (**2′**) exhibited greater antibacterial activity than compound (**1′**). The enhanced antibacterial performance of compound (**2′**) may be attributed to its distinct structural characteristics, particularly the presence of the hydroxyl substituent at the six‐position of the pyrimidine ring. Compared with 2,4‐diaminopyrimidine, the hydroxyl‐containing ligand (2,4‐diamino‐6‐hydroxypyrimidine) may increase the hydrogen‐bonding capability of the supramolecular assembly and modify its physicochemical properties, thereby promoting more favorable interactions with bacterial cell envelope components. Such structural features may also improve the aqueous dispersibility of the compound and facilitate its interaction with biological targets. Similar effects of hydroxyl‐containing ligands on the biological performance of metal‐based compounds have previously been reported [72, 73].

In addition to enhancing hydrogen‐bonding capability, hydroxyl‐containing heterocyclic ligands have been suggested to strengthen interactions with bacterial membranes through polar intermolecular contacts, thereby promoting membrane destabilization and facilitating antibacterial activity [74]. Previous investigations have further proposed that metal complexes may contribute to oxidative damage in bacterial cells through reactive oxygen species (ROS)–related pathways, although this mechanism was not directly investigated in the present study [75, 76]. In addition, adsorption of the compounds onto the bacterial membrane may locally increase membrane tension, a mechanical mechanism proposed to underlie the antibacterial action of other metal‐based nanostructures and potentially relevant to compound (**2′**) given its comparatively lower structural dimensionality [77]. Consequently, the improved antibacterial performance observed for compound (**2′**) is more reasonably attributed to the combined influence of its hydroxyl‐functionalized ligand, enhanced intermolecular interactions, and modified physicochemical properties rather than to a single structural factor. Moreover, the coordinated and lattice water molecules present in compound (**2′**) may further contribute to hydrogen‐bonding interactions within the supramolecular architecture, potentially improving molecular accessibility and dispersion in aqueous media. These combined structural characteristics may facilitate more efficient interactions with bacterial cells and consequently enhance antibacterial activity [78, 79].

The enhanced antibacterial activity of compound (**2′**) may also be associated with differences in its supramolecular organization relative to the coordination polymeric framework of compound (**1′**). Although the precise antibacterial mechanism was not investigated in the present study, previous studies have suggested that electrostatic interactions between positively polarized metal‐containing compounds and the negatively charged bacterial cell envelope may facilitate initial adhesion, thereby promoting subsequent membrane perturbation and intracellular interactions [78, 79]. In the present system, such interactions are likely influenced by the overall molecular architecture, ligand environment, and accessibility of the metal coordination sites rather than by a single structural feature. Therefore, the combined effects of the hydroxyl‐containing ligand and the distinct supramolecular arrangement of compound (**2′**) may contribute to its superior antibacterial performance [79]. The direct experimental controls using the free ligands (H_2_L, L′, and HL″) and HgCl_2_ alone were not included in the present antibacterial evaluation. Literature precedent indicates that HgCl_2_ alone exhibits broad‐spectrum antibacterial activity through binding to thiol (–SH) groups of bacterial enzymes [60], suggesting that the Hg(II) center is likely a major contributor to the antibacterial activity of compounds (**1′**) and (**2′**). Coordination to the organic ligands is expected to further modulate this activity by influencing aqueous solubility, structural stability, and Hg^2+^ bioavailability, as discussed above.

This structural distinction—namely, the discrete, nonpolymeric architecture of compound (**2′**) compared with the extended coordination polymer of compound (**1′**)—may influence the physicochemical behavior of the compounds in aqueous media. The lower structural dimensionality of compound (**2′**) may provide greater molecular mobility and improve its accessibility toward bacterial cells, thereby facilitating interactions with membrane‐associated or intracellular targets. However, these mechanistic interpretations remain speculative and require further experimental verification. Nevertheless, the present findings suggest that subtle variations in ligand functionality, supramolecular organization, and coordination environment can markedly influence the antibacterial performance of Hg(II) coordination compounds.

The distinct crystal architectures of compounds (**1′**) and (**2′**) may also contribute to the differences observed in their antibacterial activities. Compound (**1′**) forms an extended coordination polymer, whereas compound (**2′**) exists as a discrete coordination complex that assembles into a supramolecular architecture through hydrogen‐bonding and van der Waals interactions. Furthermore, the presence of the 2,4‐diamino‐6‐hydroxypyrimidine ligand in compound (**2′**) provides an additional hydroxyl functionality capable of participating in intermolecular hydrogen‐bonding interactions within the crystal structure. Although the precise relationship between crystal structure and antibacterial activity was not directly investigated in the present study, these structural characteristics may influence properties, such as aqueous accessibility, molecular flexibility, and interactions with bacterial cells, thereby contributing to the enhanced antibacterial activity observed for compound (**2′**).

Finally, it should be emphasized that the antibacterial susceptibility of different bacterial strains toward Hg(II) coordination compounds may depend on several physicochemical factors, including aqueous stability, molecular structure, coordination environment, ligand functionality, and the potential availability of biologically active Hg(II) species, in addition to the intrinsic characteristics of the bacterial strain. Various bacterial defense mechanisms, including biofilm formation, enzymatic detoxification, efflux pump activity, genetic adaptation, and alterations in membrane permeability, may influence susceptibility toward metal‐based coordination compounds [78, 79].

The inhibition zones together with the low MIC values observed in the present study suggest that the synthesized Hg(II) coordination compounds effectively suppress bacterial growth, possibly through interference with essential cellular processes, although the precise antibacterial mechanism remains to be elucidated [29].

Given the well‐documented intrinsic toxicity of mercury, the antibacterial findings reported here should be interpreted as evidence of in vitro biological activity rather than as an indication of therapeutic applicability. Furthermore, the present experimental design cannot distinguish whether the observed antibacterial effect arises from the intact Hg(II) coordination compounds or from Hg^2+^ species released through partial ligand dissociation or hydrolysis during the 24‐h incubation in aqueous culture media. Clarifying this point would require dedicated speciation studies (e.g., ICP‐MS analysis of the culture supernatant) and is proposed as a direction for future work.

We emphasize that no direct mechanistic experiments—such as intracellular ROS quantification (e.g., DCFH‐DA assay), bacterial membrane integrity/permeability assays, or measurement of Hg^2+^ release under assay conditions (e.g., by ICP‐MS)—were performed in this study. All mechanistic interpretations proposed above (ROS generation, membrane destabilization, electrostatic adhesion, and thiol‐group binding) are hypotheses formulated by analogy with previously reported metal‐based antibacterial agents, and they require dedicated experimental validation in future studies.

It should be noted that the antibacterial data above were obtained from technical duplicates within a single assay run using a common bacterial inoculum, consistent with our group’s prior methodology [23]. This approach was primarily necessitated by the limited quantity of the sonochemically synthesized powder samples, together with constraints in microbial culture resources (bacterial strains, culture media, and susceptibility‐testing reagents) and available funding. Independent biological replicates would strengthen the reproducibility of these findings and are recommended for future validation studies.

#### 3.5.2. Cytotoxicity and Selectivity Assessment by MTT Assay

This study evaluated the cytotoxic effects of Hg(II) coordination compounds (**1′**) and (**2′**) on OVCAR3 ovarian cancer cells and Vero normal cells using the MTT assay. Cell viability was evaluated over a concentration range of 0.98–1000 μg/mL, with untreated cells considered the 100% viability control. Both compounds produced concentration‐dependent effects on cell viability; however, the magnitude and profile of the response differed between OVCAR3 and Vero cells.

For compound (**1′**), Vero cells exhibited a marked reduction in viability at higher concentrations. At 1000 μg/mL, the mean cell viability was approximately 5.5%, whereas viability increased with decreasing concentration (Figure 18a). In OVCAR3 cells, compound (**1′**) produced a more pronounced reduction in cell viability, reaching approximately 0.4% at 1000 μg/mL. Cell viability progressively increased with decreasing concentration, while values slightly above the untreated control were observed at concentrations below 1.95 μg/mL. These findings indicate a substantial concentration‐dependent effect of compound (**1′**) on OVCAR3 cell viability under the experimental conditions (Figure 18b).

**FIGURE 18:**
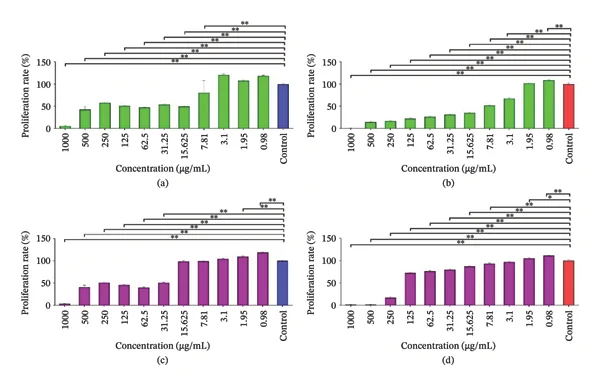
(a) The treatment of Vero normal cells and (b) OVCAR3 cells with (**1′**), (c) the treatment of Vero normal cells and (d) OVCAR3 cells with (**2′**).

Compound (**2′**) also produced marked cytotoxic effects at higher concentrations. In Vero cells, viability decreased to approximately 3.4% at 1000 μg/mL and increased progressively at lower concentrations. In OVCAR3 cells, viability was reduced to approximately 1.3% and 1.2% at 1000 and 500 μg/mL, respectively, followed by a progressive increase at lower concentrations. At concentrations below approximately 1.95 μg/mL, viability values above the untreated control were observed in both cell lines. Such increases in MTT‐derived viability may reflect altered cellular metabolic activity at low concentrations and should not, in the absence of additional mechanistic experiments, be interpreted as evidence of a biological proliferative effect (Figure 18c,d).

Overall, compounds (**1′**) and (**2′**) induced substantial loss of OVCAR3 cell viability at higher concentrations. The greater reduction in OVCAR3 viability compared with Vero cells at certain concentrations suggests differential cellular sensitivity. Importantly, the MTT assay primarily provides an assessment of cellular metabolic activity/viability and does not identify the molecular pathways responsible for the observed cytotoxic effects.

The precise mechanisms underlying the cytotoxic effects of compounds (**1′**) and (**2′**) remain to be established. Several mechanisms have been proposed for the biological activity of metal‐containing compounds, including disruption of redox homeostasis, interactions with thiol‐containing biomolecules, oxidative stress, mitochondrial dysfunction, and modulation of cell‐death pathways [80, 81]. In particular, mercury species can interact strongly with thiol‐ and selenol‐containing cellular components and may disturb cellular redox balance. However, intracellular ROS levels, oxidative stress markers, mitochondrial function, apoptosis‐related endpoints, and specific molecular targets were not directly investigated in the present study [82, 83]. Therefore, ROS generation and other proposed mechanisms should be regarded as plausible mechanistic hypotheses rather than experimentally demonstrated mechanisms for the cytotoxicity observed here [84]. Further mechanistic investigations will be required to determine the molecular basis of the observed biological effects.

In addition, the present experiments did not directly assess the stability of the coordination compounds or the extent of Hg^2+^ release or transformation under cell culture conditions. Consequently, the current data do not allow discrimination between effects caused by the intact coordination compounds and those potentially arising from released or transformed Hg‐containing species. Given the well‐established intrinsic toxicity of mercury compounds, the observed reduction in OVCAR3 cell viability should therefore be interpreted as an in vitro biological response under the experimental conditions, rather than as evidence of therapeutic potential.

To further characterize the relative sensitivity of cancerous and noncancerous cells, IC_50_ values were estimated from the concentration–response data, and selectivity indices (SI) were calculated as IC_50_(Vero)/IC_50_(OVCAR3). Because some concentration–response profiles, particularly that of compound (**2′**) in OVCAR3 cells, showed nonmonotonic behavior, the resulting IC_50_ values should be considered model‐dependent estimates of relative cellular sensitivity rather than definitive measures of cytotoxic potency or therapeutic selectivity [85, 86].

For compound (**1′**), the estimated IC_50_ values were approximately 11.37 μg/mL for Vero cells and 7.17 μg/mL for OVCAR3 cells, resulting in an SI of 1.59. This indicates a modest difference in sensitivity, with OVCAR3 cells being more sensitive to compound (**1′**) than Vero cells under the experimental conditions. In contrast, compound (**2′**) showed estimated IC_50_ values of approximately 29.25 μg/mL for Vero cells and 165.57 μg/mL for OVCAR3 cells, corresponding to an SI of 0.18. Thus, compound (**2′**) did not demonstrate preferential cytotoxicity toward OVCAR3 cells based on the calculated SI (Table 5). Taken together, compound (**1′**) showed a greater differential response between OVCAR3 and Vero cells than compound (**2′**); nevertheless, an SI of 1.59 represents only modest selectivity and should not be interpreted as evidence of strong cancer cell selectivity. The SI value of 0.18 for compound (**2′**) further indicates that its cytotoxic response was not preferential toward OVCAR3 cells. Accordingly, the IC_50_ and SI values obtained in this study provide evidence of differential cellular sensitivity rather than therapeutic selectivity [87]. This distinction is particularly important for Hg(II)‐containing compounds, for which intrinsic mercury‐associated toxicity must be considered. Further studies using additional normal and cancer cell models, together with mechanistic and Hg‐speciation analyses, are required before any conclusions regarding therapeutic potential can be drawn.

**TABLE 5:** IC_50_ values and selectivity indices (SI) of Hg(II) coordination compounds (**1′**) and (**2′**) against OVCAR3 and Vero cells.

Compound	Vero IC_50_ (μg/mL)	OVCAR3 IC_50_ (μg/mL)	SI
(**1′**)	11.37	7.17	1.59
(**2′**)	29.25	165.57	0.18

Higher SI values indicate greater relative sensitivity of OVCAR3 cells compared with Vero cells. Because the concentration–response profiles showed nonmonotonic behavior at some concentrations, the calculated IC_50_ and SI values should be interpreted as comparative indicators of cellular sensitivity rather than definitive measures of therapeutic selectivity.

## 4. Conclusion

The findings show the successful synthesis and characterization of two mercury(II) coordination compounds with different coordination environments and supramolecular structures. In the case of compound (**1**), it is anhydrous, whereas compound (**2**) contains coordinated and lattice water molecules. Both compounds are phase pure, as verified by PXRD. Topochemical models (BFDH) of compounds (**1**) and (**2**) were developed from the SCXRD data. The anisotropic growth of these specimens was mediated by the combined effect of intermolecular interactions and molecular packing (i.e., both BFDH morphologies show that most of the crystal growth occurs via slow growth of their respective crystal planes). For instance, compound (**1**) displays three slow‐growing crystal face planes: (020), (011), and (002). These crystals predominantly exhibit an elongated plate‐like morphology, reflecting slow crystal growth along the three principal planes. In contrast, compound (**2**), which is characterized by four slow‐growing crystal face planes {(011), (110), (20‐2), and (112)}, produces thin plate‐like crystals and indicates the differences between the symmetrical coordination environment of mercury(II) in compound (**1**) and the asymmetrical coordination environment of mercury(II) in compound (**2**). Crystal morphology is affected by both interplanar spacing (*d*_hkl_) and crystallographic orientation (i.e., low‐energy crystal faces). The manner in which the compound crystal forms depends upon the interactions of molecules with each other (e.g., hydrogen bonding, π‐π stacking, and dispersive forces) and interplanar spacing (*d*_hkl_), while the intermolecular interactions were analyzed using HSA. The differences between the BFDH‐predicted crystal faces obtained for both compounds and the respective PXRD data are attributable to the two methods making fundamentally different assumptions about predicted growth. About antimicrobial activity, compound (**2′**) was found to have a higher potency than (**1′**) when comparing both compounds’ MICs, providing a rationale for subsequent investigations for using (**2′**) as an antimicrobial agent in coordination compounds.

## Author Contributions

Fatemeh Jamshidi, Somayyeh Gharibi, Zeinab Asen, and Arezoo Khoradmehr: methodology and writing–original draft preparation. Khosro Mohammadi: conceptualization, supervision, writing–original draft preparation, and writing–review and editing. Payam Hayati: writing–review and editing. Jan Janczak: structural investigation and software.

## Funding

No funding was received for this manuscript.

## Conflicts of Interest

The authors declare no conflicts of interest.

## Supporting Information

Additional supporting information can be found online in the Supporting Information section.

## Supporting information


**Supporting Information** The crystallographic data for the structures in this study have been submitted to the Cambridge Crystallographic Data Centre (CCDC) as Supporting Information, under the reference numbers 2353705 and 2353564 for (**1**) and (**2**).

## Data Availability

The data that support the findings of this study are available in the supporting information of this article.
