# Supplementary material for: Mercury(II) Coordination Compounds Based on Pyridine‐2,5‐Dicarboxylic Acid: Synthesis, Crystal Structure, Morphological Characterization, and Biological Activities
Source: Bioinorg Chem Appl. 2026 Oct 29;2026:3269865. doi: 10.1155/bca/3269865 (PMC13717386; doi:10.1155/bca/3269865)
Supplement: Supplementary file 1 [file BCA-2026-3269865-s001.docx]

**Supporting Information**

**Mercury(II) Coordination Compounds Based on Pyridine-2,5-dicarboxylic Acid: Synthesis, Crystal Structure, Morphological Characterization, and Biological Activities**

Fatemeh Jamshidi^a^, Khosro Mohammadi*^a^, Payam Hayati*^b^, Somayyeh Gharibi^c^, Arezoo Khoradmehr^c^, Zeinab Asen^d^, Jan Janczak^e^

^a^ Department of Chemistry, Faculty of Nano and Bio Sciences and Technology, Persian Gulf University, Bushehr, 75169, Iran.

^b^ Department of Biotechnology and Life Sciences, University of Insubria, Via Jean Henry Dunant, 3, 21100 Varese, Italy.

^c^ The Persian Gulf Marine Biotechnology Research Center, The Persian Gulf Biomedical Research Sciences Center Institute, Bushehr University of Medical Sciences, Bushehr, 7514633196, Iran

^d^ Shamim Beh Azma Technical Inspection Company, Bushehr, Iran

^e^ Institute of Low Temperature and Structure Research, Polish Academy of Sciences, Okólna 2 Str., 50-422 Wroclaw, Poland.

Corresponding Author: Khosro Mohammadi ([khmohammadi@pgu.ac.ir](mailto:khmohammadi@pgu.ac.ir))

Table S1. The selected bond lengths (Å) and bond angles (^o^) for compounds )1( and (2)

| **Bond lengths (Å)** | | | | |  | | **Bond Angles (^o^)** | | | | | | | |  | | | |
| --- | --- | --- | --- | --- | --- | --- | --- | --- | --- | --- | --- | --- | --- | --- | --- | --- | --- | --- |
| Compound (**1**) | | | Compound (**2**) | | | | Compound (**1**) | | | Compound (**2**) | | | | | |  |  |  |
| Hg1-Cl1 | 2.356(2) | Hg1-O2 | | 2.560(6) | | Cl1-Hg1-O1 | | 93.16(2) | | | O6-Hg2-O7 | | 79.99(13) | | | |  |  |
| Hg1-Cl1i | 3.077(2) | Hg1-N1 | | 2.046(8) | | Cl1i-Hg1-O1 | | 71.60(2) | | | O7-Hg2-O6i | | 79.99(13) | | | |  |  |
| Hg1-Cl2 | 2.422(2) | Hg1-N11 | | 2.103(6) | | Cl1-Hg1-Cl1i | | 89.09(7) | | | O6i-Hg2-O1i | | 62.6(2) | | | |  |  |
| Hg1-Cl2ii | 3.287(2) | Hg1-Cl1i  Hg2-O1 | | 3.258(6)  2.855(5) | | Cl2-Hg1-N1 | | 93.22(2) | | | O1i-Hg2-O1 | | 75.2(2) | | | |  |  |
| Hg1-O1 | 2.502(8) | Hg2-O1i | | 2.855(5) | | Cl2-Hg1-Cl2ii | | 83.22(6) | | | O1-Hg2-O6 | | 62.6(2) | | | |  |  |
| Hg1-N1 | 2.364(6) | Hg2-O6 | | 2.833(6) | | N1-Hg1-Cl2ii | | 84.9(2) | | | Cl1i-Hg2-Cl1 | | 177.6(2) | | | |  |  |
| C7-O1 | 1.233(1) | Hg2-O7 | | 2.810(6) | | Cl1-Hg1-Cl2 | | 135.83(8) | | | O2-Hg1-N11 | | 72.1(2) | | | |  |  |
| C7-O2 | 1.261(1) | Hg2-O6' | | 2.833 | | Cl1-Hg1-N1 | | 125.25(2) | | | N1-Hg1-N11 | | 179.3(3) | | | |  |  |
| C8-O3 | 1.198(1) | Hg2-Cl1 | | 2.315(2) | | Cl2ii-Hg1-O1 | | 139.1(2) | | | N1-Hg1-O2 | | 108.2(3) | | | |  |  |
| C8-O4 | 1.269(1) | Hg2-Cl1i | | 2.315(2) | | **Dihedral Angles(^o^)** | |  |  | |  | |  | | | |  |  |
|  |  | Cl7-O2 | | 1.28(1) | | C9-N2-O1-C7 | | 20(1) |  | | C4-O1-C4-C3 | | 14.4 | | | |  |  |
|  |  | Cl7-O3 | | 1.25(1) | | N2-C12-O1-C7 | | 34.24(1) |  | | C7-O3-C3-H3 | | 14.18 | | | |  |  |
|  |  | Cl8-O4 | | 1.20(1) | | O1-Hg1-C12-N2 | | 11.51(5) |  | | C18-O5-N2-H2 | | | 8.53 | | | |  |
|  |  | Cl8-O5 | | 1.24(1) | | Hg1-Cl1-C12-N2 | | 33.17(6) |  | | N4-H41-H13-C13 | | 10.52 | | | |  |  |
|  |  |  | |  | | C7-O2-N4-C9 | | 17.40(9) |  | |  |  |  | | | |  |  |

**(a)**

**(b)**

**Figure S1.** (**a**) ^1^H NMR spectrum of compound (**1'**) in DMSO-d_6_. (**b**) ^13^C NMR spectrum of compound (**1'**) in DMSO-d_6_.

**Figure S2.** ^1^H NMR spectrum of compound (**2'**) in DMSO-d_6_

**Figure S3**. The FT-IR spectra of H_2_L, L′, HL′′, (**1′**) and (**2′**)

(b)

(a)

(a)

**Figure S4**. The PXRD patterns of (**a**) simulated from single crystal X-ray data of (**1**) (blue) and compound (**1'**) (red), (**b**) simulated from single crystal X-ray data of (**2**) (blue) and compound (**2'**) (red)

**(a)**

**(b)**

**Figure S5.** Crystal habit development illustrated in four panels: **a**) Stepwise inclusion of Miller-indexed planes for compound (**1**), **b**) Stepwise inclusion of Miller-indexed planes for compound (**2**), Low-index planes indicate the preferred growth direction along the *b*-axis.

**(a)**

**(b)**

**Figure S6.** EDS spectra and elemental compositions of surfaces for both compounds (**a**) (**1'**) and (**b**) (**2'**)

(a)

Distribution (%)

| Hydrodynamic diameter (nm) | qi (%) | Qi (%) |
| --- | --- | --- |
| 133.493 | 0 | 0 |
| 134.493 | 0.0164829 | 0.000303666 |
| 156.892 | 0.0755751 | 0.00161633 |
| 179.29 | 0.251182 | 0.00627561 |
| 201.689 | 0.661542 | 0.0192903 |
| 224.088 | 1.46342 | 0.0495904 |
| 246.487 | 2.82969 | 0.110807 |
| 268.886 | 4.92015 | 0.221313 |
| 291.284 | 7.85432 | 0.403466 |
| 313.683 | 11.6926 | 0.682188 |
| 336.082 | 16.4282 | 1.08317 |
| 358.481 | 21.9895 | 1.63097 |
| 380.88 | 28.25 | 2.34735 |
| 403.278 | 35.0429 | 3.24985 |
| 425.677 | 42.1776 | 4.35095 |
| 448.076 | 49.4548 | 5.65755 |
| 470.475 | 56.6804 | 7.17095 |
| 492.873 | 63.6756 | 8.88712 |
| 515.272 | 70.2845 | 10.7973 |
| 537.671 | 76.378 | 12.8886 |
| 560.07 | 81.8562 | 15.1449 |
| 582.469 | 86.6476 | 17.5476 |
| 604.867 | 90.7078 | 20.0765 |
| 627.266 | 94.0168 | 22.7105 |
| 649.665 | 96.5754 | 25.4282 |
| 672.064 | 98.4021 | 28.2084 |
| 694.463 | 99.5293 | 31.0308 |
| 716.861 | 100 | 33.8759 |
| 739.26 | 99.8649 | 36.7258 |
| 761.659 | 99.1798 | 39.564 |
| 784.058 | 98.0028 | 42.3757 |
| 806.457 | 96.3932 | 45.1476 |
| 828.855 | 94.4094 | 47.8683 |
| 851.254 | 92.1078 | 50.5278 |
| 873.653 | 89.542 | 53.118 |
| 896.052 | 86.7624 | 55.632 |
| 918.451 | 83.8151 | 58.0643 |
| 940.849 | 80.7425 | 60.4107 |
| 963.248 | 77.5827 | 62.6683 |
| 985.647 | 74.3696 | 64.835 |
| 1008.05 | 71.1333 | 66.9098 |
| 1030.44 | 67.8997 | 68.8923 |
| 1052.84 | 64.6914 | 70.7829 |
| 1075.24 | 61.5275 | 72.5827 |
| 1097.64 | 58.4241 | 74.2931 |
| 1120.04 | 55.3943 | 75.916 |
| 1142.44 | 52.449 | 77.4538 |
| 1164.84 | 49.5965 | 78.9089 |
| 1187.24 | 46.8433 | 80.284 |
| 1209.63 | 44.194 | 81.5821 |
| 1232.03 | 41.6519 | 82.8062 |
| 1254.43 | 39.2187 | 83.9594 |
| 1276.83 | 36.8951 | 85.0447 |
| 1299.23 | 34.6808 | 86.0653 |
| 1321.63 | 32.5747 | 87.0243 |
| 1344.03 | 30.575 | 87.9248 |
| 1366.43 | 28.6795 | 88.7697 |
| 1388.83 | 26.8852 | 89.562 |
| 1411.22 | 25.1893 | 90.3045 |
| 1433.62 | 23.5881 | 91.0001 |
| 1456.02 | 22.0783 | 91.6512 |
| 1478.42 | 20.6561 | 92.2606 |
| 1500.82 | 19.3177 | 92.8306 |
| 1523.22 | 18.0594 | 93.3635 |
| 1545.62 | 16.8773 | 93.8617 |
| 1568.02 | 15.7677 | 94.3272 |
| 1590.41 | 14.7268 | 94.762 |
| 1612.81 | 13.7511 | 95.1681 |
| 1635.21 | 12.8371 | 95.5472 |
| 1657.61 | 11.9812 | 95.9011 |
| 1680.01 | 11.1803 | 96.2314 |
| 1702.41 | 10.4311 | 96.5395 |
| 1724.81 | 9.73067 | 96.827 |
| 1747.21 | 9.07602 | 97.0952 |
| 1769.6 | 8.4644 | 97.3453 |
| 1792 | 7.89318 | 97.5785 |
| 1814.4 | 7.35986 | 97.796 |
| 1836.8 | 6.86206 | 97.9988 |
| 1859.2 | 6.39753 | 98.1879 |
| 1881.6 | 5.96416 | 98.3642 |
| 1904 | 5.55993 | 98.5285 |
| 1926.4 | 5.18296 | 98.6817 |
| 1948.8 | 4.83147 | 98.8245 |
| 1971.19 | 4.50379 | 98.9576 |
| 1993.59 | 4.19834 | 99.0817 |
| 2015.99 | 3.91365 | 99.1973 |
| 2038.39 | 3.64834 | 99.3052 |
| 2060.79 | 3.4011 | 99.4057 |
| 2083.19 | 3.17073 | 99.4994 |
| 2105.59 | 2.95609 | 99.5868 |
| 2127.99 | 2.75611 | 99.6682 |
| 2150.38 | 2.5698 | 99.7442 |
| 2172.78 | 2.39623 | 99.815 |
| 2195.18 | 2.23452 | 99.881 |
| 2217.58 | 2.08388 | 99.9426 |
| 2239.98 | 1.94355 | 100 |
| 2240.98 | 0 | 100 |

(b)

Distribution (%)

| Hydrodynamic diameter (nm) | qi (%) | Qi (%) |
| --- | --- | --- |
| 1010.34 | 0 | 0 |
| 1011.4 | 0.00400925 | 0.000316501 |
| 1012.46 | 0.00638276 | 0.000523787 |
| 1013.51 | 0.0100462 | 0.00085149 |
| 1014.57 | 0.0156337 | 0.00136372 |
| 1015.63 | 0.0240548 | 0.00215537 |
| 1016.69 | 0.0365961 | 0.00336515 |
| 1017.74 | 0.0550527 | 0.00519323 |
| 1018.8 | 0.0818932 | 0.00792484 |
| 1019.86 | 0.120464 | 0.0119612 |
| 1020.92 | 0.175235 | 0.0178594 |
| 1021.98 | 0.25209 | 0.0263831 |
| 1023.03 | 0.358653 | 0.0385654 |
| 1024.09 | 0.504653 | 0.0557854 |
| 1025.15 | 0.702304 | 0.0798601 |
| 1026.21 | 0.966689 | 0.113151 |
| 1027.27 | 1.31611 | 0.158685 |
| 1028.32 | 1.77237 | 0.22029 |
| 1029.38 | 2.36097 | 0.302736 |
| 1030.44 | 3.11108 | 0.411885 |
| 1031.5 | 4.05541 | 0.554832 |
| 1032.56 | 5.22967 | 0.740039 |
| 1033.61 | 6.6718 | 0.977433 |
| 1034.67 | 8.42085 | 1.27848 |
| 1035.73 | 10.5154 | 1.6562 |
| 1036.79 | 12.9918 | 2.12509 |
| 1037.85 | 15.8818 | 2.70102 |
| 1038.9 | 19.2101 | 3.40098 |
| 1039.96 | 22.9919 | 4.24277 |
| 1041.02 | 27.23 | 5.24453 |
| 1042.08 | 31.9127 | 6.42423 |
| 1043.14 | 37.0113 | 7.79903 |
| 1044.19 | 42.479 | 9.38459 |
| 1045.25 | 48.2501 | 11.1943 |
| 1046.31 | 54.2399 | 13.2387 |
| 1047.37 | 60.3462 | 15.5243 |
| 1048.43 | 66.4516 | 18.0535 |
| 1049.48 | 72.4267 | 20.8236 |
| 1050.54 | 78.1345 | 23.8268 |
| 1051.6 | 83.4356 | 27.0496 |
| 1052.66 | 88.1937 | 30.473 |
| 1053.72 | 92.2816 | 34.0729 |
| 1054.77 | 95.5866 | 37.8202 |
| 1055.83 | 98.0158 | 41.6819 |
| 1056.89 | 99.5007 | 45.6217 |
| 1057.95 | 100 | 49.6011 |
| 1059 | 99.5017 | 53.5805 |
| 1060.06 | 98.0237 | 57.5204 |
| 1061.12 | 95.6124 | 61.3828 |
| 1062.18 | 92.3407 | 65.1319 |
| 1063.24 | 88.3041 | 68.7351 |
| 1064.29 | 83.616 | 72.1643 |
| 1065.35 | 78.403 | 75.3961 |
| 1066.41 | 72.7985 | 78.412 |
| 1067.47 | 66.9379 | 81.1993 |
| 1068.53 | 60.9528 | 83.7503 |
| 1069.58 | 54.9668 | 86.0625 |
| 1070.64 | 49.0912 | 88.1381 |
| 1071.7 | 43.4228 | 89.9835 |
| 1072.76 | 38.0411 | 91.6084 |
| 1073.82 | 33.0084 | 93.0256 |
| 1074.87 | 28.3688 | 94.2499 |
| 1075.93 | 24.1501 | 95.2974 |
| 1076.99 | 20.3642 | 96.1853 |
| 1078.05 | 17.0098 | 96.9308 |
| 1079.11 | 14.0743 | 97.5508 |
| 1080.16 | 11.5362 | 98.0617 |
| 1081.22 | 9.36743 | 98.4786 |
| 1082.28 | 7.53546 | 98.8158 |
| 1083.34 | 6.00541 | 99.0859 |
| 1084.4 | 4.74168 | 99.3003 |
| 1085.45 | 3.70929 | 99.4688 |
| 1086.51 | 2.87494 | 99.6002 |
| 1087.57 | 2.20779 | 99.7015 |
| 1088.63 | 1.67993 | 99.7791 |
| 1089.69 | 1.2666 | 99.8379 |
| 1090.74 | 0.946273 | 99.882 |
| 1091.8 | 0.700537 | 99.9149 |
| 1092.86 | 0.513921 | 99.9391 |
| 1093.92 | 0.373615 | 99.9568 |
| 1094.98 | 0.269169 | 99.9696 |
| 1096.03 | 0.192181 | 99.9788 |
| 1097.09 | 0.135986 | 99.9854 |
| 1098.15 | 0.0953639 | 99.99 |
| 1099.21 | 0.0662817 | 99.9932 |
| 1100.26 | 0.0456598 | 99.9954 |
| 1101.32 | 0.0311758 | 99.997 |
| 1102.38 | 0.0210986 | 99.998 |
| 1103.44 | 0.0141532 | 99.9987 |
| 1104.5 | 0.00941085 | 99.9992 |
| 1105.55 | 0.00620286 | 99.9995 |
| 1106.61 | 0.00405277 | 99.9997 |
| 1107.67 | 0.00262496 | 99.9998 |
| 1108.73 | 0 | 100 |

**Figure S7**. Hydrodynamic particle size distributions obtained by dynamic light scattering (DLS) for (**a**) compound (**1′**) and (**b**) compound (**2′**)

**(a)**

**(b)**

% Weight loss

Heat Flow Endo Up (mW)

| Temperature (°C) | Weight % (%) | Heat Flow Endo Up (mW) |
| --- | --- | --- |
| 48.41 | 100.038 | 11.6686 |
| 50.41 | 100.211 | 11.0483 |
| 52.41 | 100.269 | 10.3626 |
| 54.41 | 100.307 | 9.93433 |
| 56.41 | 100.338 | 9.63333 |
| 58.41 | 100.366 | 9.40857 |
| 60.41 | 100.381 | 9.24497 |
| 62.41 | 100.404 | 9.11347 |
| 64.41 | 100.424 | 9.01037 |
| 66.41 | 100.437 | 8.92937 |
| 68.41 | 100.442 | 8.8599 |
| 70.41 | 100.457 | 8.80573 |
| 72.41 | 100.472 | 8.75623 |
| 74.41 | 100.477 | 8.71547 |
| 76.41 | 100.487 | 8.68227 |
| 78.41 | 100.496 | 8.65413 |
| 80.41 | 100.505 | 8.62473 |
| 82.41 | 100.517 | 8.604 |
| 84.41 | 100.528 | 8.58213 |
| 86.41 | 100.541 | 8.55663 |
| 88.41 | 100.557 | 8.53693 |
| 90.41 | 100.556 | 8.5083 |
| 92.41 | 100.559 | 8.48667 |
| 94.41 | 100.561 | 8.46853 |
| 96.41 | 100.574 | 8.44277 |
| 98.41 | 100.581 | 8.41933 |
| 100.41 | 100.584 | 8.38847 |
| 102.41 | 100.594 | 8.35907 |
| 104.41 | 100.604 | 8.32773 |
| 106.41 | 100.609 | 8.2955 |
| 108.41 | 100.617 | 8.2588 |
| 110.41 | 100.622 | 8.2224 |
| 112.41 | 100.627 | 8.18813 |
| 114.41 | 100.635 | 8.14693 |
| 116.41 | 100.641 | 8.1098 |
| 118.41 | 100.647 | 8.06757 |
| 120.41 | 100.65 | 8.02527 |
| 122.41 | 100.658 | 7.98073 |
| 124.41 | 100.664 | 7.9293 |
| 126.41 | 100.67 | 7.8811 |
| 128.41 | 100.675 | 7.82467 |
| 130.41 | 100.68 | 7.7725 |
| 132.41 | 100.683 | 7.74283 |
| 134.41 | 100.683 | 7.7074 |
| 136.41 | 100.68 | 7.67397 |
| 138.41 | 100.678 | 7.63157 |
| 140.41 | 100.68 | 7.59093 |
| 142.41 | 100.697 | 7.53827 |
| 144.41 | 100.701 | 7.48807 |
| 146.41 | 100.701 | 7.43213 |
| 148.41 | 100.7 | 7.37003 |
| 150.41 | 100.701 | 7.30573 |
| 152.41 | 100.698 | 7.2326 |
| 154.41 | 100.693 | 7.16597 |
| 156.41 | 100.693 | 7.0939 |
| 158.41 | 100.693 | 7.016 |
| 160.41 | 100.691 | 6.93837 |
| 162.41 | 100.688 | 6.8548 |
| 164.41 | 100.688 | 6.771 |
| 166.41 | 100.688 | 6.6849 |
| 168.41 | 100.683 | 6.59597 |
| 170.41 | 100.679 | 6.50567 |
| 172.41 | 100.678 | 6.4171 |
| 174.41 | 100.673 | 6.33173 |
| 176.41 | 100.665 | 6.2434 |
| 178.41 | 100.667 | 6.15107 |
| 180.41 | 100.666 | 6.0602 |
| 182.41 | 100.661 | 5.96947 |
| 184.41 | 100.654 | 5.8793 |
| 186.41 | 100.645 | 5.79143 |
| 188.41 | 100.627 | 5.69497 |
| 190.41 | 100.617 | 5.6097 |
| 192.41 | 100.609 | 5.51627 |
| 194.41 | 100.599 | 5.4236 |
| 196.41 | 100.589 | 5.33317 |
| 198.41 | 100.566 | 5.23637 |
| 200.41 | 100.548 | 5.15377 |
| 202.41 | 100.533 | 5.07283 |
| 204.41 | 100.515 | 4.9751 |
| 206.41 | 100.492 | 4.88367 |
| 208.41 | 100.472 | 4.7942 |
| 210.41 | 100.443 | 4.69973 |
| 212.41 | 100.404 | 4.61767 |
| 214.41 | 100.367 | 4.53483 |
| 216.41 | 100.338 | 4.45233 |
| 218.41 | 100.3 | 4.3735 |
| 220.41 | 100.254 | 4.2905 |
| 222.41 | 100.203 | 4.21857 |
| 224.41 | 100.155 | 4.1441 |
| 226.41 | 100.089 | 4.07103 |
| 228.41 | 100.015 | 4.0072 |
| 230.41 | 99.931 | 3.9401 |
| 232.41 | 99.84 | 3.87703 |
| 234.41 | 99.74 | 3.81757 |
| 236.41 | 99.633 | 3.76897 |
| 238.41 | 99.485 | 3.71693 |
| 240.41 | 99.34 | 3.67383 |
| 242.41 | 99.172 | 3.63813 |
| 244.41 | 98.959 | 3.6147 |
| 246.41 | 98.74 | 3.60077 |
| 248.41 | 98.481 | 3.59347 |
| 250.41 | 98.172 | 3.58923 |
| 252.41 | 97.829 | 3.60697 |
| 254.41 | 97.399 | 3.64017 |
| 256.41 | 96.915 | 3.69393 |
| 258.41 | 96.338 | 3.78397 |
| 260.41 | 95.653 | 3.9144 |
| 262.41 | 94.824 | 4.119 |
| 264.41 | 93.823 | 4.41413 |
| 266.41 | 92.587 | 4.85517 |
| 268.41 | 91.023 | 5.53137 |
| 270.41 | 88.994 | 6.61323 |
| 272.41 | 86.126 | 8.72503 |
| 274.41 | 81.471 | 12.2299 |
| 276.41 | 73.382 | 15.8051 |
| 278.41 | 66.607 | 13.0268 |
| 280.41 | 61.257 | 10.67 |
| 282.41 | 56.914 | 8.74987 |
| 284.41 | 53.252 | 7.23917 |
| 286.41 | 50.25 | 5.9996 |
| 288.41 | 47.825 | 5.00293 |
| 290.41 | 45.784 | 4.3133 |
| 292.41 | 43.942 | 3.89873 |
| 294.41 | 42.246 | 3.72727 |
| 296.41 | 40.635 | 3.7062 |
| 298.41 | 39.044 | 3.7804 |
| 300.41 | 37.487 | 3.86093 |
| 302.41 | 35.944 | 3.92073 |
| 304.41 | 34.443 | 3.94597 |
| 306.41 | 32.986 | 3.9171 |
| 308.41 | 31.606 | 3.81863 |
| 310.41 | 30.331 | 3.65353 |
| 312.41 | 29.179 | 3.4322 |
| 314.41 | 28.22 | 3.0932 |
| 316.41 | 27.415 | 2.71527 |
| 318.41 | 26.759 | 2.39873 |
| 320.41 | 26.166 | 2.1855 |
| 322.41 | 25.634 | 2.05047 |
| 324.41 | 25.176 | 1.90837 |
| 326.41 | 24.752 | 1.80503 |
| 328.41 | 24.374 | 1.7445 |
| 330.41 | 23.979 | 1.71193 |
| 332.41 | 23.591 | 1.69433 |
| 334.41 | 23.251 | 1.6906 |
| 336.41 | 22.876 | 1.7298 |
| 338.41 | 22.493 | 1.8026 |
| 340.41 | 22.056 | 1.88813 |
| 342.41 | 21.716 | 1.93697 |
| 344.41 | 21.378 | 1.90603 |
| 346.41 | 21.066 | 1.91693 |
| 348.41 | 20.774 | 1.89893 |
| 350.41 | 20.498 | 1.92333 |
| 352.41 | 20.225 | 1.96433 |
| 354.41 | 19.921 | 2.03083 |
| 356.41 | 19.639 | 2.06003 |
| 358.41 | 19.395 | 2.0727 |
| 360.41 | 19.141 | 2.0936 |
| 362.41 | 18.907 | 2.1198 |
| 364.41 | 18.68 | 2.1602 |
| 366.41 | 18.466 | 2.1932 |
| 368.41 | 18.265 | 2.22977 |
| 370.41 | 18.064 | 2.2724 |
| 372.41 | 17.87 | 2.32957 |
| 374.41 | 17.679 | 2.37753 |
| 376.41 | 17.499 | 2.4293 |
| 378.41 | 17.326 | 2.48523 |
| 380.41 | 17.152 | 2.54523 |
| 382.41 | 17.012 | 2.61147 |
| 384.41 | 16.838 | 2.6745 |
| 386.41 | 16.702 | 2.74607 |
| 388.41 | 16.557 | 2.822 |
| 390.41 | 16.417 | 2.90153 |
| 392.41 | 16.301 | 2.98803 |
| 394.41 | 16.172 | 3.07517 |
| 396.41 | 16.048 | 3.16673 |
| 398.41 | 15.925 | 3.2617 |
| 400.41 | 15.811 | 3.35847 |
| 402.41 | 15.709 | 3.4617 |
| 404.41 | 15.6 | 3.56337 |
| 406.41 | 15.509 | 3.6686 |
| 408.41 | 15.424 | 3.77773 |
| 410.41 | 15.346 | 3.8897 |
| 412.41 | 15.275 | 4.00663 |
| 414.41 | 15.209 | 4.12607 |
| 416.41 | 15.151 | 4.24737 |
| 418.41 | 15.091 | 4.37693 |
| 420.41 | 15.036 | 4.51113 |
| 422.41 | 14.981 | 4.64623 |
| 424.41 | 14.935 | 4.7843 |
| 426.41 | 14.897 | 4.92283 |
| 428.41 | 14.854 | 5.0741 |
| 430.41 | 14.805 | 5.22757 |
| 432.41 | 14.765 | 5.3841 |
| 434.41 | 14.724 | 5.53707 |
| 436.41 | 14.689 | 5.69507 |
| 438.41 | 14.645 | 5.85577 |
| 440.41 | 14.612 | 6.0263 |
| 442.41 | 14.587 | 6.19503 |
| 444.41 | 14.544 | 6.36383 |
| 446.41 | 14.519 | 6.5334 |

|  | Derivative Weight % (%/m |
| --- | --- |
| 48.41 | -0.002708 |
| 50.41 | 0.245 |
| 52.41 | 0.164 |
| 54.41 | 0.154 |
| 56.41 | 0.151 |
| 58.41 | 0.112 |
| 60.41 | 0.106 |
| 62.41 | 0.129 |
| 64.41 | 0.097 |
| 66.41 | 0.057 |
| 68.41 | 0.067 |
| 70.41 | 0.103 |
| 72.41 | 0.078 |
| 74.41 | 0.049 |
| 76.41 | 0.064 |
| 78.41 | 0.063 |
| 80.41 | 0.072 |
| 82.41 | 0.081 |
| 84.41 | 0.086 |
| 86.41 | 0.096 |
| 88.41 | 0.057 |
| 90.41 | 0.0005448 |
| 92.41 | 0.013 |
| 94.41 | 0.053 |
| 96.41 | 0.065 |
| 98.41 | 0.044 |
| 100.41 | 0.055 |
| 102.41 | 0.067 |
| 104.41 | 0.056 |
| 106.41 | 0.048 |
| 108.41 | 0.055 |
| 110.41 | 0.046 |
| 112.41 | 0.04 |
| 114.41 | 0.052 |
| 116.41 | 0.052 |
| 118.41 | 0.04 |
| 120.41 | 0.033 |
| 122.41 | 0.048 |
| 124.41 | 0.056 |
| 126.41 | 0.044 |
| 128.41 | 0.033 |
| 130.41 | 0.035 |
| 132.41 | 0.009573 |
| 134.41 | -0.008067 |
| 136.41 | -0.02 |
| 138.41 | 0.013 |
| 140.41 | 0.064 |
| 142.41 | 0.062 |
| 144.41 | 0.014 |
| 146.41 | -0.002071 |
| 148.41 | 0.00756 |
| 150.41 | -0.00999 |
| 152.41 | -0.029 |
| 154.41 | -0.016 |
| 156.41 | -3.344e-08 |
| 158.41 | -0.016 |
| 160.41 | -0.023 |
| 162.41 | -0.008016 |
| 164.41 | 0.002448 |
| 166.41 | -0.022 |
| 168.41 | -0.039 |
| 170.41 | -0.025 |
| 172.41 | -0.025 |
| 174.41 | -0.04 |
| 176.41 | -0.019 |
| 178.41 | -0.004537 |
| 180.41 | -0.017 |
| 182.41 | -0.051 |
| 184.41 | -0.072 |
| 186.41 | -0.098 |
| 188.41 | -0.093 |
| 190.41 | -0.074 |
| 192.41 | -0.065 |
| 194.41 | -0.088 |
| 196.41 | -0.123 |
| 198.41 | -0.14 |
| 200.41 | -0.136 |
| 202.41 | -0.137 |
| 204.41 | -0.157 |
| 206.41 | -0.169 |
| 208.41 | -0.203 |
| 210.41 | -0.248 |
| 212.41 | -0.298 |
| 214.41 | -0.264 |
| 216.41 | -0.279 |
| 218.41 | -0.32 |
| 220.41 | -0.354 |
| 222.41 | -0.385 |
| 224.41 | -0.456 |
| 226.41 | -0.548 |
| 228.41 | -0.603 |
| 230.41 | -0.659 |
| 232.41 | -0.735 |
| 234.41 | -0.798 |
| 236.41 | -0.953 |
| 238.41 | -1.125 |
| 240.41 | -1.232 |
| 242.41 | -1.459 |
| 244.41 | -1.619 |
| 246.41 | -1.874 |
| 248.41 | -2.168 |
| 250.41 | -2.459 |
| 252.41 | -2.868 |
| 254.41 | -3.393 |
| 256.41 | -3.989 |
| 258.41 | -4.679 |
| 260.41 | -5.545 |
| 262.41 | -6.723 |
| 264.41 | -8.068 |
| 266.41 | -9.921 |
| 268.41 | -12.324 |
| 270.41 | -15.826 |
| 272.41 | -21.635 |
| 274.41 | -33.291 |
| 276.41 | -48.767 |
| 278.41 | -48.626 |
| 280.41 | -40.447 |
| 282.41 | -33.756 |
| 284.41 | -28.036 |
| 286.41 | -23.015 |
| 288.41 | -18.935 |
| 290.41 | -16.165 |
| 292.41 | -14.359 |
| 294.41 | -13.238 |
| 296.41 | -12.557 |
| 298.41 | -12.216 |
| 300.41 | -11.827 |
| 302.41 | -11.647 |
| 304.41 | -11.327 |
| 306.41 | -10.834 |
| 308.41 | -10.151 |
| 310.41 | -9.269 |
| 312.41 | -8.177 |
| 314.41 | -6.904 |
| 316.41 | -5.717 |
| 318.41 | -4.903 |
| 320.41 | -4.315 |
| 322.41 | -3.843 |
| 324.41 | -3.389 |
| 326.41 | -3.099 |
| 328.41 | -2.951 |
| 330.41 | -2.898 |
| 332.41 | -2.788 |
| 334.41 | -2.779 |
| 336.41 | -2.891 |
| 338.41 | -3.058 |
| 340.41 | -2.895 |
| 342.41 | -2.578 |
| 344.41 | -2.416 |
| 346.41 | -2.291 |
| 348.41 | -2.15 |
| 350.41 | -2.097 |
| 352.41 | -2.17 |
| 354.41 | -2.143 |
| 356.41 | -1.985 |
| 358.41 | -1.894 |
| 360.41 | -1.817 |
| 362.41 | -1.745 |
| 364.41 | -1.689 |
| 366.41 | -1.592 |
| 368.41 | -1.559 |
| 370.41 | -1.472 |
| 372.41 | -1.439 |
| 374.41 | -1.378 |
| 376.41 | -1.357 |
| 378.41 | -1.301 |
| 380.41 | -1.203 |
| 382.41 | -1.192 |
| 384.41 | -1.152 |
| 386.41 | -1.047 |
| 388.41 | -1.056 |
| 390.41 | -0.991 |
| 392.41 | -0.978 |
| 394.41 | -0.933 |
| 396.41 | -0.896 |
| 398.41 | -0.847 |
| 400.41 | -0.8 |
| 402.41 | -0.756 |
| 404.41 | -0.71 |
| 406.41 | -0.643 |
| 408.41 | -0.625 |
| 410.41 | -0.559 |
| 412.41 | -0.513 |
| 414.41 | -0.454 |
| 416.41 | -0.437 |
| 418.41 | -0.421 |
| 420.41 | -0.393 |
| 422.41 | -0.363 |
| 424.41 | -0.319 |
| 426.41 | -0.311 |
| 428.41 | -0.336 |
| 430.41 | -0.327 |
| 432.41 | -0.304 |
| 434.41 | -0.293 |
| 436.41 | -0.305 |
| 438.41 | -0.269 |
| 440.41 | -0.227 |
| 442.41 | -0.251 |
| 444.41 | -0.249 |
| 446.41 | -0.222 |

% Weight loss

Heat Flow Endo Up (mW)

| Temperature (°C) | Weight % (%) | Heat Flow Endo Up (mW) |
| --- | --- | --- |
| 47.73 | 99.581 | 12.0005 |
| 49.73 | 99.6 | 11.7855 |
| 51.73 | 99.695 | 11.5831 |
| 53.73 | 99.686 | 11.4627 |
| 55.73 | 99.686 | 11.3797 |
| 57.73 | 99.686 | 11.3219 |
| 59.73 | 99.676 | 11.2799 |
| 61.73 | 99.648 | 11.2488 |
| 63.73 | 99.591 | 11.2263 |
| 65.73 | 99.562 | 11.2092 |
| 67.73 | 99.496 | 11.1986 |
| 69.73 | 99.405 | 11.1925 |
| 71.73 | 99.334 | 11.1905 |
| 73.73 | 99.248 | 11.191 |
| 75.73 | 99.172 | 11.1934 |
| 77.73 | 99.096 | 11.1981 |
| 79.73 | 99.01 | 11.2047 |
| 81.73 | 98.905 | 11.2094 |
| 83.73 | 98.782 | 11.2148 |
| 85.73 | 98.677 | 11.2203 |
| 87.73 | 98.534 | 11.2252 |
| 89.73 | 98.391 | 11.2289 |
| 91.73 | 98.244 | 11.2317 |
| 93.73 | 98.125 | 11.2321 |
| 95.73 | 98.001 | 11.2304 |
| 97.73 | 97.887 | 11.2292 |
| 99.73 | 97.782 | 11.2272 |
| 101.73 | 97.649 | 11.2239 |
| 103.73 | 97.535 | 11.2192 |
| 105.73 | 97.43 | 11.2131 |
| 107.73 | 97.344 | 11.2035 |
| 109.73 | 97.259 | 11.1926 |
| 111.73 | 97.221 | 11.1807 |
| 113.73 | 97.183 | 11.1691 |
| 115.73 | 97.135 | 11.1584 |
| 117.73 | 97.097 | 11.1477 |
| 119.73 | 97.068 | 11.1371 |
| 121.73 | 97.059 | 11.1256 |
| 123.73 | 97.04 | 11.1145 |
| 125.73 | 97.04 | 11.1051 |
| 127.73 | 97.03 | 11.0972 |
| 129.73 | 97.002 | 11.089 |
| 131.73 | 96.973 | 11.0836 |
| 133.73 | 96.964 | 11.0788 |
| 135.73 | 96.954 | 11.0763 |
| 137.73 | 96.926 | 11.0723 |
| 139.73 | 96.916 | 11.0672 |
| 141.73 | 96.926 | 11.0595 |
| 143.73 | 96.926 | 11.0522 |
| 145.73 | 96.897 | 11.0426 |
| 147.73 | 96.878 | 11.0308 |
| 149.73 | 96.878 | 11.0198 |
| 151.73 | 96.868 | 11.0074 |
| 153.73 | 96.88 | 10.9936 |
| 155.73 | 96.868 | 10.9801 |
| 157.73 | 96.849 | 10.9687 |
| 159.73 | 96.83 | 10.953 |
| 161.73 | 96.835 | 10.9362 |
| 163.73 | 96.821 | 10.9201 |
| 165.73 | 96.786 | 10.901 |
| 167.73 | 96.792 | 10.8832 |
| 169.73 | 96.783 | 10.8652 |
| 171.73 | 96.754 | 10.8476 |
| 173.73 | 96.726 | 10.8288 |
| 175.73 | 96.697 | 10.809 |
| 177.73 | 96.669 | 10.7923 |
| 179.73 | 96.65 | 10.7739 |
| 181.73 | 96.63 | 10.7548 |
| 183.73 | 96.592 | 10.7357 |
| 185.73 | 96.552 | 10.7165 |
| 187.73 | 96.519 | 10.6977 |
| 189.73 | 96.478 | 10.677 |
| 191.73 | 96.44 | 10.6577 |
| 193.73 | 96.374 | 10.6378 |
| 195.73 | 96.316 | 10.6198 |
| 197.73 | 96.25 | 10.601 |
| 199.73 | 96.155 | 10.5808 |
| 201.73 | 96.097 | 10.5627 |
| 203.73 | 96.031 | 10.5451 |
| 205.73 | 95.936 | 10.5251 |
| 207.73 | 95.798 | 10.5083 |
| 209.73 | 95.65 | 10.4912 |
| 211.73 | 95.555 | 10.4746 |
| 213.73 | 95.422 | 10.4563 |
| 215.73 | 95.269 | 10.438 |
| 217.73 | 95.108 | 10.4202 |
| 219.73 | 94.96 | 10.4022 |
| 221.73 | 94.784 | 10.3843 |
| 223.73 | 94.603 | 10.3666 |
| 225.73 | 94.356 | 10.3497 |
| 227.73 | 94.175 | 10.3332 |
| 229.73 | 93.877 | 10.3181 |
| 231.73 | 93.581 | 10.3012 |
| 233.73 | 93.328 | 10.2833 |
| 235.73 | 92.975 | 10.2695 |
| 237.73 | 92.576 | 10.2558 |
| 239.73 | 92.219 | 10.2426 |
| 241.73 | 91.817 | 10.23 |
| 243.73 | 91.31 | 10.221 |
| 245.73 | 90.829 | 10.21 |
| 247.73 | 90.196 | 10.2047 |
| 249.73 | 89.555 | 10.198 |
| 251.73 | 88.819 | 10.1901 |
| 253.73 | 88.016 | 10.1812 |
| 255.73 | 87.166 | 10.1722 |
| 257.73 | 86.167 | 10.1626 |
| 259.73 | 85.066 | 10.1547 |
| 261.73 | 83.857 | 10.1483 |
| 263.73 | 82.477 | 10.1412 |
| 265.73 | 81.144 | 10.1366 |
| 267.73 | 79.589 | 10.132 |
| 269.73 | 77.893 | 10.1283 |
| 271.73 | 76.16 | 10.1268 |
| 273.73 | 74.243 | 10.1234 |
| 275.73 | 72.28 | 10.1228 |
| 277.73 | 70.135 | 10.1171 |
| 279.73 | 67.98 | 10.1079 |
| 281.73 | 65.8 | 10.0986 |
| 283.73 | 63.647 | 10.087 |
| 285.73 | 61.635 | 10.0765 |
| 287.73 | 59.512 | 10.0663 |
| 289.73 | 57.428 | 10.0579 |
| 291.73 | 55.352 | 10.0492 |
| 293.73 | 53.322 | 10.0367 |
| 295.73 | 51.347 | 10.0224 |
| 297.73 | 49.549 | 10.0004 |
| 299.73 | 47.935 | 9.97252 |
| 301.73 | 46.495 | 9.94574 |
| 303.73 | 45.246 | 9.9188 |
| 305.73 | 44.254 | 9.8951 |
| 307.73 | 43.328 | 9.87627 |
| 309.73 | 42.601 | 9.86342 |
| 311.73 | 41.864 | 9.85029 |
| 313.73 | 41.162 | 9.84303 |
| 315.73 | 40.529 | 9.83737 |
| 317.73 | 39.891 | 9.83467 |
| 319.73 | 39.313 | 9.8313 |
| 321.73 | 38.771 | 9.83025 |
| 323.73 | 38.175 | 9.82599 |
| 325.73 | 37.607 | 9.82738 |
| 327.73 | 37.055 | 9.82728 |
| 329.73 | 36.493 | 9.82859 |
| 331.73 | 35.96 | 9.83197 |
| 333.73 | 35.437 | 9.83492 |
| 335.73 | 34.847 | 9.83827 |
| 337.73 | 34.253 | 9.84408 |
| 339.73 | 33.638 | 9.84858 |
| 341.73 | 33.107 | 9.85495 |
| 343.73 | 32.517 | 9.86024 |
| 345.73 | 31.972 | 9.86856 |
| 347.73 | 31.417 | 9.8767 |
| 349.73 | 30.906 | 9.88485 |
| 351.73 | 30.364 | 9.89437 |
| 353.73 | 29.969 | 9.90417 |
| 355.73 | 29.44 | 9.91437 |
| 357.73 | 29.022 | 9.92488 |
| 359.73 | 28.65 | 9.93581 |
| 361.73 | 28.279 | 9.94858 |
| 363.73 | 28.013 | 9.95785 |
| 365.73 | 27.759 | 9.97242 |
| 367.73 | 27.537 | 9.98614 |
| 369.73 | 27.311 | 9.99965 |
| 371.73 | 27.127 | 10.0174 |
| 373.73 | 26.95 | 10.0348 |
| 375.73 | 26.842 | 10.0528 |
| 377.73 | 26.709 | 10.0712 |
| 379.73 | 26.556 | 10.0908 |
| 381.73 | 26.461 | 10.1115 |
| 383.73 | 26.325 | 10.1334 |
| 385.73 | 26.185 | 10.1565 |
| 387.73 | 26.09 | 10.1796 |
| 389.73 | 25.963 | 10.2034 |
| 391.73 | 25.842 | 10.2271 |
| 393.73 | 25.709 | 10.2537 |
| 395.73 | 25.633 | 10.2793 |
| 397.73 | 25.528 | 10.3071 |
| 399.73 | 25.412 | 10.334 |
| 401.73 | 25.281 | 10.3642 |
| 403.73 | 25.205 | 10.3934 |
| 405.73 | 25.109 | 10.4245 |
| 407.73 | 25.024 | 10.4547 |
| 409.73 | 24.906 | 10.4878 |
| 411.73 | 24.795 | 10.5189 |
| 413.73 | 24.719 | 10.5527 |
| 415.73 | 24.634 | 10.5866 |
| 417.73 | 24.529 | 10.6212 |
| 419.73 | 24.396 | 10.659 |
| 421.73 | 24.319 | 10.6943 |
| 423.73 | 24.212 | 10.7309 |
| 425.73 | 24.101 | 10.7688 |
| 427.73 | 24.027 | 10.811 |
| 429.73 | 23.929 | 10.8523 |
| 431.73 | 23.796 | 10.8919 |
| 433.73 | 23.691 | 10.9342 |
| 435.73 | 23.587 | 10.9777 |
| 437.73 | 23.482 | 11.0236 |
| 439.73 | 23.377 | 11.0682 |
| 441.73 | 23.215 | 11.1132 |
| 443.73 | 23.12 | 11.1599 |
| 445.73 | 23.015 | 11.2069 |

|  | Derivative Weight % (%/m |
| --- | --- |
| 47.73 | 17.011 |
| 49.73 | 10.437 |
| 51.73 | 10.152 |
| 53.73 | 9.908 |
| 55.73 | 10.086 |
| 57.73 | 9.971 |
| 59.73 | 9.901 |
| 61.73 | 9.703 |
| 63.73 | 9.719 |
| 65.73 | 9.684 |
| 67.73 | 9.493 |
| 69.73 | 9.47 |
| 71.73 | 9.485 |
| 73.73 | 9.425 |
| 75.73 | 9.44 |
| 77.73 | 9.418 |
| 79.73 | 9.33 |
| 81.73 | 9.23 |
| 83.73 | 9.167 |
| 85.73 | 9.128 |
| 87.73 | 8.935 |
| 89.73 | 8.984 |
| 91.73 | 9.067 |
| 93.73 | 9.1 |
| 95.73 | 9.105 |
| 97.73 | 9.189 |
| 99.73 | 9.093 |
| 101.73 | 9.114 |
| 103.73 | 9.183 |
| 105.73 | 9.255 |
| 107.73 | 9.365 |
| 109.73 | 9.513 |
| 111.73 | 9.692 |
| 113.73 | 9.697 |
| 115.73 | 9.669 |
| 117.73 | 9.759 |
| 119.73 | 9.845 |
| 121.73 | 9.916 |
| 123.73 | 9.939 |
| 125.73 | 9.95 |
| 127.73 | 9.849 |
| 129.73 | 9.809 |
| 131.73 | 9.881 |
| 133.73 | 9.907 |
| 135.73 | 9.831 |
| 137.73 | 9.877 |
| 139.73 | 9.985 |
| 141.73 | 10.027 |
| 143.73 | 9.899 |
| 145.73 | 9.835 |
| 147.73 | 9.905 |
| 149.73 | 9.99995 |
| 151.73 | 10.058 |
| 153.73 | 10.0001 |
| 155.73 | 9.885 |
| 157.73 | 9.874 |
| 159.73 | 9.958 |
| 161.73 | 9.948 |
| 163.73 | 9.845 |
| 165.73 | 9.892 |
| 167.73 | 9.946 |
| 169.73 | 9.855 |
| 171.73 | 9.771 |
| 173.73 | 9.815 |
| 175.73 | 9.818 |
| 177.73 | 9.824 |
| 179.73 | 9.82 |
| 181.73 | 9.773 |
| 183.73 | 9.708 |
| 185.73 | 9.724 |
| 187.73 | 9.75 |
| 189.73 | 9.695 |
| 191.73 | 9.606 |
| 193.73 | 9.52 |
| 195.73 | 9.501 |
| 197.73 | 9.395 |
| 199.73 | 9.449 |
| 201.73 | 9.501 |
| 203.73 | 9.389 |
| 205.73 | 9.124 |
| 207.73 | 8.963 |
| 209.73 | 9.049 |
| 211.73 | 9.068 |
| 213.73 | 8.934 |
| 215.73 | 8.778 |
| 217.73 | 8.857 |
| 219.73 | 8.734 |
| 221.73 | 8.57 |
| 223.73 | 8.307 |
| 225.73 | 8.192 |
| 227.73 | 8.161 |
| 229.73 | 7.794 |
| 231.73 | 7.838 |
| 233.73 | 7.667 |
| 235.73 | 7.214 |
| 237.73 | 7.131 |
| 239.73 | 6.973 |
| 241.73 | 6.422 |
| 243.73 | 6.009 |
| 245.73 | 5.839 |
| 247.73 | 5.324 |
| 249.73 | 4.775 |
| 251.73 | 4.233 |
| 253.73 | 3.558 |
| 255.73 | 2.835 |
| 257.73 | 2.185 |
| 259.73 | 1.274 |
| 261.73 | 0.395 |
| 263.73 | -0.3 |
| 265.73 | -1.076 |
| 267.73 | -1.981 |
| 269.73 | -2.915 |
| 271.73 | -3.718 |
| 273.73 | -4.49 |
| 275.73 | -5.335 |
| 277.73 | -5.893 |
| 279.73 | -6.058 |
| 281.73 | -6.108 |
| 283.73 | -5.74 |
| 285.73 | -5.703 |
| 287.73 | -5.703 |
| 289.73 | -5.503 |
| 291.73 | -5.625 |
| 293.73 | -5.016 |
| 295.73 | -4.344 |
| 297.73 | -3.179 |
| 299.73 | -1.451 |
| 301.73 | 0.142 |
| 303.73 | 1.641 |
| 305.73 | 2.665 |
| 307.73 | 3.61 |
| 309.73 | 4.349 |
| 311.73 | 4.723 |
| 313.73 | 5.047 |
| 315.73 | 5.259 |
| 317.73 | 5.301 |
| 319.73 | 5.631 |
| 321.73 | 5.724 |
| 323.73 | 5.734 |
| 325.73 | 5.798 |
| 327.73 | 5.796 |
| 329.73 | 5.859 |
| 331.73 | 5.958 |
| 333.73 | 5.801 |
| 335.73 | 5.617 |
| 337.73 | 5.477 |
| 339.73 | 5.514 |
| 341.73 | 5.719 |
| 343.73 | 5.724 |
| 345.73 | 5.938 |
| 347.73 | 5.937 |
| 349.73 | 6.023 |
| 351.73 | 6.277 |
| 353.73 | 6.482 |
| 355.73 | 6.536 |
| 357.73 | 7.101 |
| 359.73 | 7.371 |
| 361.73 | 7.618 |
| 363.73 | 7.981 |
| 365.73 | 8.258 |
| 367.73 | 8.369 |
| 369.73 | 8.561 |
| 371.73 | 8.675 |
| 373.73 | 8.885 |
| 375.73 | 8.951 |
| 377.73 | 9.017 |
| 379.73 | 9.002 |
| 381.73 | 9.104 |
| 383.73 | 9.031 |
| 385.73 | 9.09 |
| 387.73 | 9.152 |
| 389.73 | 9.093 |
| 391.73 | 9.096 |
| 393.73 | 9.236 |
| 395.73 | 9.263 |
| 397.73 | 9.171 |
| 399.73 | 9.108 |
| 401.73 | 9.175 |
| 403.73 | 9.344 |
| 405.73 | 9.341 |
| 407.73 | 9.232 |
| 409.73 | 9.17 |
| 411.73 | 9.308 |
| 413.73 | 9.328 |
| 415.73 | 9.318 |
| 417.73 | 9.169 |
| 419.73 | 9.205 |
| 421.73 | 9.278 |
| 423.73 | 9.222 |
| 425.73 | 9.323 |
| 427.73 | 9.334 |
| 429.73 | 9.179 |
| 431.73 | 9.146 |
| 433.73 | 9.193 |
| 435.73 | 9.206 |
| 437.73 | 9.185 |
| 439.73 | 9.026 |
| 441.73 | 9.074 |
| 443.73 | 9.25 |
| 445.73 | 9.281 |

**Figure S8.** Thermal behavior of (**a**) (**1'**) and (**b**) (**2'**)

(**a**)

(**b**)

**Figure S9.** Crystal morphology of (**a**) (**1**) and (**b**) (**2**) predicted by the BFDH method

(**a**)

(**b**)

**Figure S10.** Crystal structures of (**a**) (**1**) and (**b**) (**2**) with the dominant planes (highest relative areas) indicated
